# In Vitro Fermentation of Green Tea by Human Gut Microbiota Enhances Bioactivity and Bidirectionally Modulates Polyphenol Metabolites and Gut Microbiota

**DOI:** 10.3390/foods15101732

**Published:** 2026-05-14

**Authors:** Kaiyin Hu, Jinxin Liu, You Su, Yijun Wang, Huimin Guo, Xiaochun Wan, Zhongwen Xie, Li Sun

**Affiliations:** 1 The College of Pharmacy, Anhui University of Chinese Medicine, Hefei 230012, China; kaiyinhuaht@163.com (K.H.); liujxwyyx@163.com (J.L.); suyouahu@163.com (Y.S.); 2 State Key Laboratory of Tea Plant Germplasm Innovation and Utilization, School of Tea Sciences, Anhui Agricultural University, Hefei 230036, China; yjwang@ahau.edu.cn (Y.W.); xcwan@ahau.edu.cn (X.W.); 3 Center for Biotechnology, Anhui Agricultural University, Hefei 230036, China; huiminguo@ahau.edu.cn

**Keywords:** green tea, gut microbial metabolism, bioactive metabolites, flavonoid metabolic pathway, polyphenol biotransformation, untargeted metabolomics

## Abstract

Green tea is highly popular due to its richness in polyphenols exhibiting broad bioactivities. Tea polyphenols, primarily catechins and flavonoids, demonstrate health benefits following biotransformation by the gut microbiota to overcome limited bioavailability. However, metabolites and interaction between green tea polyphenol and the gut microbiota remains to be fully elucidated. This study investigates the biotransformation of metabolites and interaction between human gut microbiota (HGM) and green tea extract (GTE) through in vitro anaerobic fermentation. Temporal bioactivity assessments demonstrated that fermentation-enhanced antioxidant capacity and inhibition potential of *α*-glucosidase, *α*-amylase and pancreatic lipase peak at 6 h, showing strong correlations with polyphenol and flavonoid biotransformation kinetics. Using the untargeted metabolomics approach, 55 characteristic differential compounds during the fermentation process in GTE were characterized, including 15 catechins, 29 flavonoids, five organic acids and six other phytochemicals. Furthermore, nine microbial-transformed metabolites derived from GTE flavonoids were identified and the corresponding metabolic pathways were proposed simultaneously. Analysis of 16S rRNA gene sequencing revealed that GTE significantly enhanced gut microbiota diversity and induced structural reorganization, specifically enriching genera such as *Bacteroides*, *Bifidobacterium*, *Lactococcus* and *Enterococcus*, which are likely involved in flavonoid biotransformation of GTE. Thus, the findings provide new insights for elucidating microbiota-mediated metabolites of green tea polyphenol, and their bidirectional interactions in the human gut.

## 1. Introduction

Tea is derived from the processed leaves and buds of a tea plant (*Camellia sinensis* (L.) Kuntze.) through specialized agricultural practices and manufacturing techniques [1,2]. The growing body of evidence from prospective cohort studies suggests that regular tea consumption demonstrates potential preventive effects against multiple chronic disorders [3]. Notably, epidemiological investigations have identified an inverse association between sustained tea intake and the incidence of irritable bowel syndrome, along with cerebrovascular pathologies including ischemic stroke and vascular dementia [3,4]. Furthermore, specific population studies indicate that habitual green tea consumption may confer protective benefits against metabolic disorders exemplified by type 2 diabetes mellitus (T2DM) and its associated complications [5].

Green tea contains a diverse array of phytochemicals that mediate its broad pharmacological effects. A myriad of clinical evidence demonstrates that green tea bioactive compounds can ameliorate non-alcoholic fatty liver disease (NAFLD) by modulating intestinal lipid absorption and hepatic lipid metabolism, while concurrently improving metabolic dysregulation in glucose and lipid homeostasis [6,7]. Contemporary research has further validated its multi-target therapeutic potential, revealing cytoprotective properties encompassing anti-neoplastic, anti-inflammatory, and anti-aging effects, complemented by immunoregulatory capacities and organoprotective actions on cardiovascular and gastrointestinal systems [8,9,10,11,12]. The health benefits principally originate from green tea polyphenols, particularly catechins (epicatechin derivatives) and flavonol-type antioxidants that constitute the primary bioactive fraction [13,14]. These compounds share a characteristic diphenylpropane backbone (C6-C3-C6 configuration), manifesting as either flavan-3-ol or 2-phenylchromen-4-one derivatives depending on oxygenation patterns and ring cyclization [15,16]. Critical to their bioactivity, the spatial arrangement and hydroxylation status of substituent groups on this core structure dictate molecular interactions with biological targets, with even minor stereochemical variations significantly altering therapeutic efficacy [15,17,18]. This structure–function relationship suggests that chemical modifications of tea flavonoids could strategically enhance their pharmacological profiles, though the current understanding of such structure–activity correlations remains incomplete, necessitating systematic structure–activity relationship analyses in future investigations.

The human gut microbiome can be considered as an involute and manifold microecosystem encompassing bacteria, archaea, eukaryotes, and viruses that symbiotically colonize gastrointestinal mucosa [19,20]. Emerging evidence indicates that ingested tea components undergo extensive biotransformation through the microbial metabolism, generating bioactive metabolites with modified physicochemical properties and enhanced bioavailability [21]. Crucially, intestinal microbiota governs polyphenol pharmacokinetics. Current data suggest merely 5–10% of dietary polyphenols achieve direct absorption in the proximal gut, with the majority being catabolized by colonic microbiota into low-molecular-weight derivatives [22,23]. This enteric microbial processing involves sequential enzymatic modifications (e.g., dehydroxylation, hydrogenation, ring cleavage) that may yield structurally novel polyphenolic metabolites with unique biological activities [24]. As principal dietary sources of polyphenols, green tea derivatives undergo this microbial metabolic reprogramming, potentially amplifying their therapeutic potential through enteric conversion. Importantly, the microbial metabolites may exhibit distinct bioactivity profiles compared to parental compounds, potentially mediating tea’s systemic health effects via the gut–brain axis and enteroendocrine signaling pathways. However, little is known about what and how tea compounds, especially polyphenols, metabolize by microbiota in the human intestinal gut. Addressing these key knowledge gaps will advance precision nutrition strategies leveraging tea–microbiota crosstalk for targeted health interventions.

The in vitro anaerobic fermentation method has consistently been one of the most practical and well-established approaches for studying the metabolism of dietary polyphenols by colonic microbiota, widely applied in the analysis and identification of metabolites derived from polyphenols such as catechins, theaflavins and other polyphenols from various food sources [25]. Thus, to investigate gut microbiota-mediated biotransformation of green tea phytochemicals, we employed an in vitro colonic fermentation model under anaerobic conditions. Temporal monitoring of bioactive component metabolism was achieved through metabolomic profiling based on Ultra-High-Performance Liquid Chromatography coupled with Quadrupole-Orbitrap Tandem Mass Spectrometry (UHPLC-Q-Orbitrap-MS/MS), complemented by antioxidant capacity and enzymatic inhibition assays. Multivariate analysis integrating temporal functional variations with differential metabolite identification revealed microbial metabolic conversion patterns affecting tea bioactivity. Through this integrative approach, we sought to uncover both the dynamic biotransformation of green tea polyphenols by the human gut microbiota and the corresponding functional implications for their bioactivity, providing insight into the microbiota-mediated modulation of green tea extracts in the human gastrointestinal tract.

## 2. Materials and Methods

### 2.1. Chemicals and Reagents

Standard chemical epigallocatechin (EGC), epicatechin gallate (ECG), epigallocatechin gallate (EGCG), epicatechin (EC), all with a purity of 98%, were purchased from Chengdu Munster Biotechnology Co., Ltd. (Chengdu, China); quercetin-3-O-rutinoside, quercetin, quercetin-3-O-galactoside and gallic acid, 3-phenylpropionic acid, phenylacetic acid, 3-(3,4-dihydroxyphenyl) propionic acid, *p*-hydroxybenzoic acid and other phenolic acids (at least 98% purities), as well as pancreatic lipase (porcine) and *p*-nitrophenyl-*α*-D-glucopyranoside (pNP-G), were obtained from Yuanye Biotechnology Co., Ltd. (Shanghai, China). 2,2′-azino-bis (3-Ethylbenzothiazoline-6-sulfonic acid) (ABTS) and 2,2-diphenyl-1-picrylhydrazyl (DPPH) were purchased from Merck Life Sciences Co., Ltd. (Sigma-Aldrich, St. Louis, MO, USA). *α*-glucosidase, *α*-amylase, 3,5-dinitrosalicylic acid (DNS), GAM broth, vitamin K1, cysteine, hemin and acarbose were purchased from Solarbio Life Sciences Co., Ltd. (Beijing, China); 4-nitrophenyl laurate (p-NPL), Folin–Ciocalteu’s phenol reagent, and phosphate-buffered saline (PBS) were purchased from Macklin Biotechnology Co., Ltd. (Shanghai, China); Rutin, trolox, 2,4,6-tri(2-Pyridyl)-1,3,5-triazine (TPTZ), iron chloride hexahydrate and L-ascorbic acid were purchased from Aladdin Biotechnology Co., Ltd. (Shanghai, China). Cholestyramine was purchased from MedChemExpress (Monmouth Junction, NJ, USA).

### 2.2. Sample Preparation and Collection

Huangshan Maofeng green tea was purchased from Huangshan Maofeng Tea Group Company (Huangshan, China), and processed through optimized aqueous extraction. The tea powder (1:20 *w*/*v*) underwent thermostatically agitated boiling water infusion (20 min) using a C-MAG HS 7 magnetic stirrer (IKA, Staufen, Germany), followed by ultrasonication (15 min) with a KQ-500DE ultrasonic cleaner (Kunshan Ultrasonic Instruments Co., Ltd., Kunshan, China). After centrifugation at 8000× *g* for 10 min using a centrifuge (Eppendorf, Hamburg, Germany), the supernatants were vacuum-filtered and concentrated using an RV10 rotary evaporator (IKA, Staufen, Germany), and then lyophilized with a FreeZone 4.5 freeze dryer (Labconco, Kansas City, MO, USA) to obtain standardized GTE for subsequent analyses.

Fecal inocula were prepared from eight Chinese donors balanced by gender (20–25-year-olds, BMI 18.5–23.5 kg/m^2^) meeting strict inclusion criteria: no tea consumption for at least 14 d, antibiotic-free for at least 3 months, and abstinence from smoking or alcohol. Following aseptic aliquoting of individual samples, representative 1 g portions were anaerobically homogenized in sterile DPBS (0.1 M, pH 6.9) to create a 10% (*w*/*v*) microbiota slurry. Sequential filtration through 4-layer sterile gauze, and differential centrifugation at 500× *g* for 5 min, yielded standardized human gut microbiota inoculum (HGMI), with donor characteristics detailed in Table S1.

### 2.3. Anaerobic Fermentation of GTE via Human Gut Microbiota In Vitro

In this experiment, the in vitro anaerobic fermentation procedure was adapted from previous studies with slight modifications [26]. HGMI (5 mL) was aseptically combined with 40 mL GAM broth in serum bottles, establishing primary anaerobic cultures at 37 °C with 5% CO_2_ and 95% N_2_ in a CO_2_ incubator (NU-5500E, New Air, Plymouth, MN, USA). Following 12 h microbial activation, cultures were supplemented with a 5 mL GTE solution (10 mg/mL in ultrapure water) for an additional 48 h fermentation. During the fermentation process, 1 mL of aliquot was collected at defined intervals of 0, 3, 6, 12, 24, 48 h, and was immediately quenched with 0.2 mL pre-cooled acetonitrile (4 °C). Metabolite profiling supernatants obtained through refrigerated centrifugation (12,000× *g*, 10 min, 4 °C) underwent UHPLC-MS/MS analysis, while parallel samples preserved at −80 °C enabled 16S rDNA sequencing of temporal microbiota dynamics. Vehicle controls received equivalent ultrapure water substitution. Biological triplicate samples ensured experimental validity.

### 2.4. UHPLC-Q-Orbitrap-MS/MS Analysis of Fermented Green Tea Metabolites

Time series fermented samples were homogenized with three volumes of pre-chilled acetonitrile (−20 °C). After triple-phase extraction via centrifugation at 12,000× *g* for 10 min at 4 °C, pooled supernatants were filtered through 0.22 μm PTFE membranes (MilliporeSigma, Merck KGaA, Darmstadt, Germany) prior to UHPLC-MS/MS analysis. The extraction protocol was optimized per established methodologies [27]. Chromatographic separation was achieved on an Acquity HSS T3 column (50 × 2.1 mm, 1.8 μm, Waters, Milford, MA, USA) maintained at 40 °C with 0.2 mL/min flow rate. The mobile phases used for elution were A (0.075% formic acid aqueous solution) and B (acetonitrile, LC-MS grade) and the elution gradient followed the procedure: 0–2 min, 99% A; 2–22 min, 99–1% A; 22–28 min, 1% A; 28–30 min, 99% A. Post-run equilibration for 5 min ensured system stability.

The Thermo Fisher Q-Exactive Focus Orbitrap ultra-high-resolution mass spectrometer (Thermo Fisher Scientific, Waltham, MA, USA) was employed for the mass spectrometric detection and analysis of metabolites. The mass spectrometer was operated in both positive and negative modes with data-dependent acquisition (DDA), employing full-scan MS (*m*/*z* 150–1200) for comprehensive metabolite profiling. High-purity nitrogen served as nebulizing (35 arb), auxiliary (10 arb), and collision gas. Ionization parameters including capillary voltage (±3.5 kV) and desolvation temperature (320 °C) were optimized as per manufacturer specifications. Raw MS data containing precursor/fragment ion information were processed through Compound Discoverer (version 2.1) for molecular formula assignment and fragmentation pattern matching. An integrated analytical workflow in Xcalibur (version 3.1, Thermo Fisher Scientific, Waltham, MA, USA) enabled feature alignment, peak integration, and abundance normalization across samples.

### 2.5. Antioxidant Assay

The DPPH and ABTS assays were carried out following previous protocols with minor modifications [28,29]. Antioxidant capacity was quantified using standardized radical scavenging assays. Briefly, for DPPH analysis, 250 μL of sample solutions reacted with a 750 μL pre-calibrated DPPH working solution at 37 °C for 30 min under dark; absorbance was measured at 517 nm. Additionally, an ABTS assay involved pre-generation of radicals by K_2_S_2_O_8_ activation for 16 h under dark, followed by a 6 min chromogenic reaction with test samples at 37 °C. Control 1 uses methanol substitution for the working solution, and Control 2 sets methanol substitution for the sample solution, with corresponding absorbance measurements serving as baseline references. Both assays employed ascorbic acid controls (0.01 mg/mL) and quantified radical scavenging activity following manufacture protocol. All reactions were conducted in triplicate using 96-well microplates (SpectraMax, Shanghai Megu Molecular Instruments Co., Ltd., Shanghai, China) and a microplate reader with spectrophotometric detection (SpectraMax i3x, Molecular Devices, LLC, Sunnyvale, CA, USA).

The total antioxidant capacity (T-AOC) was quantitatively determined using the ferric reducing antioxidant power (FRAP) assay [30]. A trolox standard solution (1.59 mg/mL) was initially prepared in anhydrous ethanol and serially diluted to establish a calibration curve (5.0–30.0 μg/mL). The FRAP working solution consisted of 20 mM FeCl_3_, 10 mM TPTZ, and 0.3 M acetate buffer (pH 3.6) mixed at 1:1:10 (*v*/*v*/*v*). A mixture containing 750 μL of a FRAP working solution, 150 μL of deionized water, and 100 μL of trolox standard was incubated at 37 °C in the dark for 10 min, followed by spectrophotometric measurement at 593 nm. T-AOC of the sample solution was expressed as trolox equivalents (TE) per milliliter of green tea fermentation liquid (GE, TE/mL GE).

### 2.6. Measurement of α-Glucosidase, α-Amylase and Pancreatic Lipase Inhibitory Capacity

The in vitro inhibitory capacities of *α*-glucosidase and *α*-amylase were measured based on previously reported methods with some modifications [31]. In brief, *α*-glucosidase (0.1 U/mL) and *α*-amylase (1.0 U/mL) solutions were prepared in PBS (pH 7.0). For *α*-glucosidase inhibition, 50 μL fermented tea samples and 100 μL enzyme solution were pre-incubated at 37 °C for 10 min before adding 2.5 mM pNP-G substrate. The mixture was incubated at 37 °C for 15 min under dark, and absorbance was measured at 405 nm. For the *α*-amylase inhibition assay, pre-incubated sample–enzyme mixtures (1:1 *v*/*v*) were reacted with 1% soluble starch (85 °C, 20 min pre-treatment) at 37 °C for 15 min. Reactions were terminated by adding 300 μL of DNS reagent at 100 °C for 10 min, and were then cooled, diluted to 1 mL with PBS, and were measured at 540 nm. Negative controls replaced enzyme solutions with PBS, while blank controls contained PBS in lieu of both enzyme and sample solutions. Acarbose with the concentration of 0.08 mg/mL served as the reference inhibitor. Enzyme inhibition rates were calculated by following manufacture protocols. All experiments were performed in triplicate to ensure methodological reproducibility.

The pancreatic lipase assay was adapted from the study by Li et al. with slight modifications [32]. Pancreatic lipase at the concentration of 10 mg/mL was reconstituted in deionized water, vortexed, and centrifuged at 12,000× *g* for 10 min to collect the supernatant. *p*-Nitrophenyl laurate (p-NPL, 0.8 mg·mL^−1^) substrate was prepared in sodium acetate buffer (5 mM, 1% Triton X-100) with heating at 100 °C for 5 min. Reaction mixtures containing 150 μL lipase supernatant, 50 μL samples, and 350 μL Tris-HCl (50 mM, pH 8.0) were pre-equilibrated at 37 °C for 10 min. After adding 450 μL of the substrate, mixtures were incubated at 37 °C for 2 h. Absorbance of centrifuged supernatants (12,000× *g*, 10 min) was measured at 405 nm. Negative controls substituted enzyme and sample solutions with Tris buffer, while blank controls contained only the buffer matrix. Colestyramine at a concentration of 0.02 mg/mL served as the reference inhibitor. Triplicate measurements ensured analytical reliability.

### 2.7. Determination of Total Polyphenol and Total Flavonoid Content

The Folin–Ciocalteu colorimetric method was implemented following the Chinese national standard GB/T 31740.2-2015 [33]. Gallic acid (GA) standard of a 1.0 mg/mL concentration was diluted to 10–60 μg/mL. Samples and standards (0.5 mL, respectively) reacted with a 5 mL 10% Folin–Ciocalteu reagent for 6 min in dark, followed by 4.5 mL 7.5% Na_2_CO_3_. After 60 min of dark incubation at an ambient temperature, absorbance was measured at 765 nm against ultrapure water blanks. Total polyphenol content was calculated via GA standard curve linear regression (R^2^ > 0.99) and expressed as gallic acid equivalents per mL (GAE/mL). Methodological reproducibility was ensured through triplicate independent measurements.

Quantitative determination of total flavonoid content was employed with protocol optimization based on established methodologies [34]. Rutin standards (2 mg/mL in 70% methanol) were diluted to 20–120 μg/mL. The 100 μL samples or standards were mixed with 400 μL 70% methanol and 30 μL 5% NaNO_2_ in microcentrifuge tubes. After 5 min at ambient temperature, 30 μL 10% AlCl_3_ was added to the mixture. Following 6 min of incubation at 37 °C, 200 μL of 1 M NaOH was introduced with volume adjustment to 1 mL using 70% methanol. Post 30 min incubation at 37 °C, 200 μL aliquots underwent spectrophotometry at 510 nm against 70% methanol blanks. Total flavonoids were calculated via rutin standard curve (R^2^ > 0.99), expressed as rutin equivalents/mL (RE/mL). Triplicate technical replicates ensured analytical precision.

### 2.8. Metataxonomic Profiling of In Vitro Gut Microbiota

Fermentation time series samples were centrifuged at 12,000× *g* for 10 min at 4 °C to pellet microbial biomass. Supernatants were discarded and the pellets stored at −80 °C pending nucleic acid extraction. Genomic DNA was isolated using the DNA Kit (Transgen Biotech, Beijing, China), with quality verification via agarose gel electrophoresis and Qubit 3.0 Spectrophotometer (Thermo Fisher Scientific, Waltham, MA, USA). The V3-V4 hypervariable regions were amplified with Phusion High-Fidelity DNA Polymerase using universal primers 341F/805R, incorporating Illumina sequencing adapters. The target amplicon was the 16S V3-V4 region, with primers set as 341F (5′-CCTACGGGNG GCWGCAG-3′) and 805R (5′-GACTACHVGGGTATCTAATCC-3′). Positive controls containing a microbial community standard DNA mix were processed in parallel. Library fragment distribution (120–200 bp) was validated on an ABI 2720 Thermal Cycler (Applied Biosystems, Foster City, CA, USA). Quantification via Qubit dsDNA HS Assay preceded equimolar pooling for Illumina NovaSeq 6000 sequencing (2 × 250 bp paired-end, Illumina, San Diego, CA, USA) performed by Genesky Biotechnologies Inc., Shanghai, China. Raw reads were demultiplexed using Quantitative Insights Into Microbial Ecology (QIIME 2), followed by Divisive Amplicon Denoising Algorithm 2 (DADA2)-based denoising for amplicon sequence variants (ASVs) identification. Taxonomic assignment of ASVs against the SILVA 138.1 database was performed with a pre-trained Naive Bayes classifier. The resulting data were subjected to bioinformatics analysis.

### 2.9. Data Processing and Analysis

All experimental data were compiled in Microsoft Excel and preprocessed prior to statistical analysis conducted in SPSS (version 26.0). Continuous variables are expressed as mean ± SEM. Intergroup comparisons were performed using Student’s *t*-test for two-group analyses and one-way ANOVA for multi-group comparisons. Additionally, the principal component analysis (PCA), as well as partial least squares discrimination analysis (PLS-DA) and its Hotelling’s T2 test (*p* < 0.05) were both performed in SIMCA 14.1 (MKS Umetrics, Umea, Sweden). Statistical significance was defined as *p* < 0.05. Final data visualization was created using GraphPad Prism (version 8.0.2).

UHPLC-MS/MS data acquisition and analysis were conducted using Thermo Xcalibur (version 3.0), with compound annotation performed through Compound Discoverer (version 2.1). Raw data were processed using XCMS for peak detection, alignment, and retention time correction, followed by total ion current (TIC) normalization and RSD-based filtration (quality controls with RSD > 30%) to eliminate technical variability, with subsequent feature table refinement through blank subtraction and missing value imputation prior to multivariate statistical analysis. Integrated online data acquisition coupled with multivariate data analysis enabled comprehensive characterization of human gut microbiota-mediated metabolites derived from GTE. Structural elucidation of tea metabolites was supported by established flavonoid fragmentation patterns documented in previous studies [26,35]. The analytical workflow includes the following strategies: (1) Data acquisition: Full-scan MS coupled with data-dependent MS^2^ acquisition generated high-resolution spectra. Compound Discoverer 2.1 facilitated automated fragment ion selection and preliminary metabolite annotation through integrated spectral interpretation algorithms. (2) Signal refinement: Raw data underwent sequential two-stage processing: primary screening employing mass defect filtering with dynamic background subtraction (MDF-DBS) to remove non-target interference, followed by secondary characterization through MSn-guided chromatographic extraction for molecular weight or formula determination, augmented by diagnostic product ions (DPIs) and neutral loss fragments (NLFs) for phytochemical categorization. (3) Metabolite annotation: Mass spectrometry information annotation was achieved through database matching, biotransformation-guided structural elucidation of flavonoid derivatives, and isomer discrimination via ChemDraw 14.0-predicted Clog *p* combined with RT/MS^2^ alignment against reference standards. (4) Multivariate analysis: VIP score ranked datasets (SIMCA-P 14.0) integrated with Mann–Whitney U-test and fold change (FC) filtering (VIP > 1.0, *p* < 0.05, FC < 0.5/> 2.0). Finally, MetaboAnalyst 5.0-processed data (PQN-normalized) underwent PCA/PLS-DA modeling analysis.

## 3. Results

### 3.1. Dynamic Alterations in α-Glucosidase, α-Amylase, Pancreatic Lipase Inhibitory Activities, Antioxidant Capacity, and Polyphenol–Flavonoid Content of GTE During Microbial Fermentation

**Figure 1:**
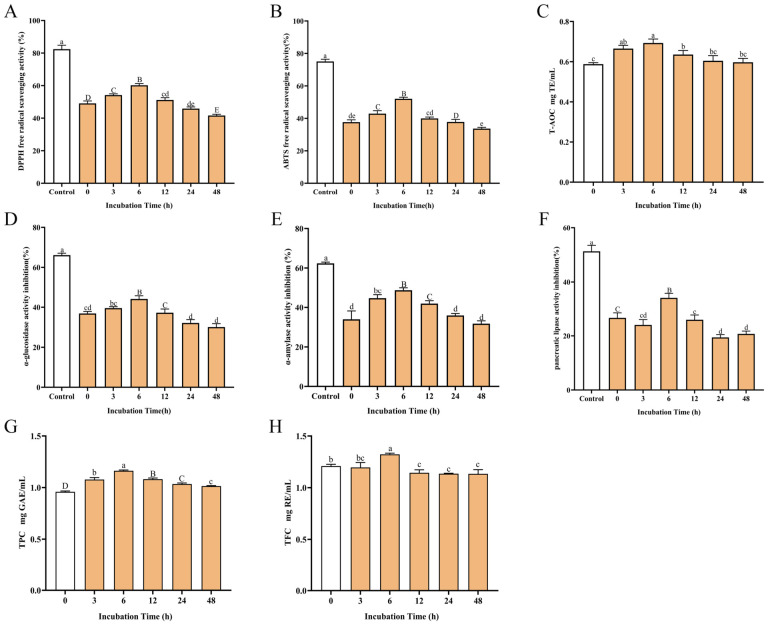
Changes in antioxidant activities, digestive enzyme inhibitory activities, and phenolic contents during incubation at time points of 0, 3, 6, 12, 24, and 48 h. 2,2-diphenyl-1-picrylhydrazyl (DPPH) free radical scavenging activity (**A**), 2,2′-azino-bis(3-ethylbenzothiazoline-6-sulfonic acid) (ABTS) free radical scavenging activity (**B**), total antioxidant capacity (T-AOC) (**C**), *α*-glucosidase inhibitory activity (**D**), *α*-amylase inhibitory activity (**E**), pancreatic lipase inhibitory activity (**F**), total phenolic content (TPC) (**G**), and total flavonoid content (TFC) (**H**) are represented by vertical bars. The presence of different letters above group columns indicates significant differences (*n* = 3, *p* < 0.05).

To monitor fermentation-dependent changes in GTE’s antioxidant activity during in vitro HGMI metabolism, we evaluated DPPH and ABTS radical scavenging capacities at six temporal points. As shown in Figure 1A,B, antioxidant activity displayed fermentation time-dependent modulation, peaking at 6 h fermentation with significant enhancements versus initial time point (0 h), 3 h, and 12 h groups (*p* < 0.05), and post-6 h fermentation showed progressive activity decline with reduced inter-group differentiation. Notwithstanding methodological variations, the antioxidant findings demonstrate enhanced bioactivity of GTE constituents through gut microbiota fermentation, with peak efficacy particularly evident at the 6 h time point. Subsequent FRAP-based T-AOC quantification confirmed maximum antioxidant capacity in 6 h fermented samples (Figure 1C and Figure S1A). The complete data is shown in Table S2. These consistent patterns across complementary assays demonstrate time-dependent optimization of microbial fermentation for enhancing green tea antioxidants.

As key carbohydrate-digesting enzymes, *α*-glucosidase and *α*-amylase drive postprandial hyperglycemia through starch hydrolysis, making their pivotal inhibitor targets for T2DM therapeutics via delayed monosaccharide release and competitive inhibition [36]. The inhibitory effects of GTE at various fermentation durations on *α*-glucosidase, *α*-amylase, and pancreatic lipase activities were evaluated via substrate-specific colorimetric assays. As shown in Figure 1D,E, compared to initial time point controls (0 h), HGMI-fermented green tea showed progressive enhancement of *α*-glucosidase and *α*-amylase inhibition, peaking at 6 h fermentation with 44.13% and 48.70% inhibition respectively. Parallel analysis revealed analogous fermentation-dependent inhibition of lipid-metabolizing pancreatic lipase (Figure 1F), with maximal suppression (34.02%) coinciding with peak carbohydrate enzyme inhibition. In any case, these results indicate that after HGMI fermentation, GTE metabolites significantly enhanced the inhibitory capacities of these three enzymes over time. Furthermore, the activity modulation pattern, characterized by peak efficacy at 6 h with subsequent attenuation, suggests coordinated biotransformation of GTE’s bioactive constituents during microbial fermentation, potentially regulating carbohydrate and lipid digestive pathways through synchronized mechanisms.

Polyphenols are regarded as main functional compounds in green tea. Polyphenols (flavonoids and catechins) were quantitatively profiled during the fermentation process using validated colorimetric assays. Calibration curves demonstrated excellent linearity (Polyphenols: Y = 0.005477X + 0.03942, R^2^ = 0.9995; Flavonoids: Y = 0.0005393X − 0.002011, R^2^ = 0.9984; Figures S1B,C). The results showed that polyphenol content peaked at 6 h (1.1626 mg GAE/mL, *p* < 0.05; Figure 1G) and maintained sustained elevation across all fermented groups, while flavonoid content showed transient optimization (6 h maximum: 1.3222 mg RE/mL) followed by significant decrease at 12 h (*p* < 0.05 versus 0 h group) (Figure 1H). The content changes at these different fermentation times may indicate that microbially mediated biotransformation preferentially stabilizes polyphenolic structures while accelerating flavonoid catabolism, peaking at 6 h. Our results indicate that the contents of polyphenols are closely correlated to antioxidant activity and the enzymes’ inhibitory potential.

### 3.2. Characteristic Metabolites in GTE Fermented by Gut Microbiota In Vitro

**Figure 2:**
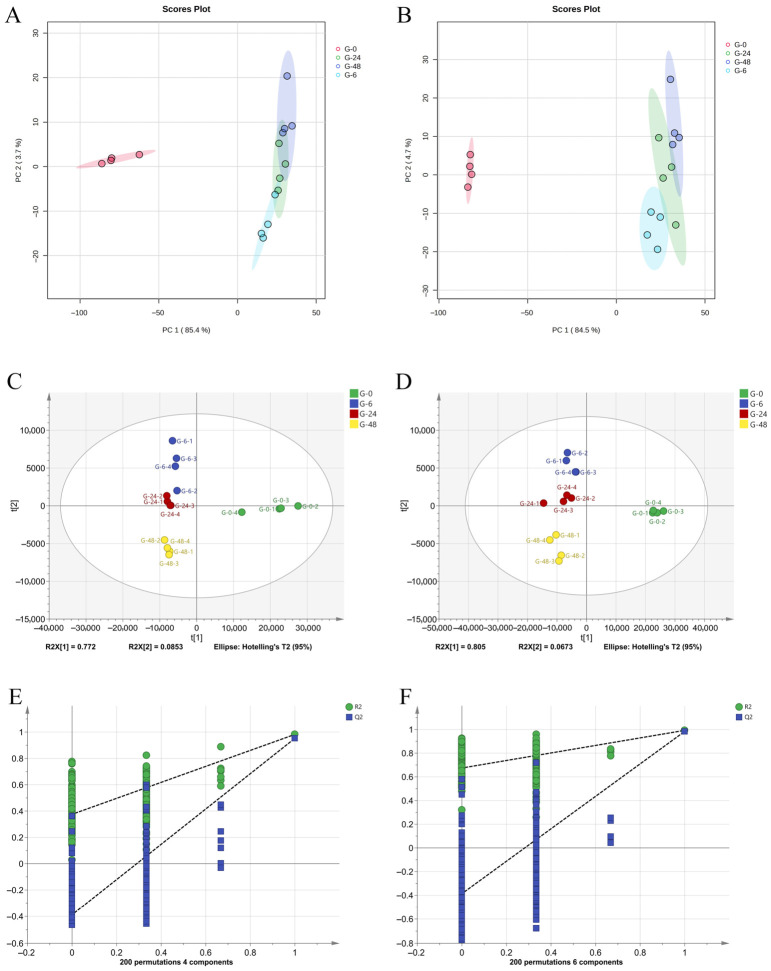
PCA score plots and PLS-DA analysis of UHPLC-Orbitrap-MS/MS data of GTE samples at different fermentation times. PCA score plot in negative ion mode (**A**); PCA score plot in positive ion mode (**B**); PLS-DA analysis in negative ion mode (**C**); PLS-DA analysis in positive ion mode (**D**); permutation test model for data analysis in negative ion mode (**E**); permutation test model for data analysis in positive ion mode (**F**).

Chromatographic separation achieved comprehensive resolution of GTE metabolites through alternating positive and negative ionization modes by UHPLC-MS/MS approaches. Total ion chromatogram (TIC) of unfermented and various fermentation time of GTE samples are displayed in Figures S2 and S3, respectively. Eventually, 2898 and 2539 qualified ion features were screened as differential metabolites in positive and negative ion modes respectively, which were then imported into MetaboAnalyst 5.0 for subsequent PCA and PLS-DA multivariate analyses, as detailed in Figure 2. Initial unsupervised PCA demonstrated distinct separation between 0 h unfermented samples and fermented groups in both ionization modes. In addition, there is relative separation among 6 h, 24 h and 48 h fermented samples. These results indicate microbial fermentation-induced dynamic alterations in metabolic profiles of GTE. Quality control samples formed tight clusters with clear segregation from experimental groups, confirming analytical stability and inter-group differentiation (Figure 2A,B). Furthermore, PLS-DA models effectively discriminated fermented samples at 6 h, 24 h and 48 h along PC1 in both ionization modes, showing favorable model parameters (positive mode: R^2^Y = 0.981, Q^2^ = 0.888; negative mode: R^2^Y = 0.950, Q^2^ = 0.798) (Figure 2C,D). Permutation validation confirmed model robustness with R^2^ intercepts of 0.675 (positive) and 0.375 (negative), and Q^2^ intercepts below −0.38, demonstrating absence of overfitting (Figure 2E,F). Subsequently, OPLS-DA analysis of 6 h fermented versus unfermented groups revealed significant metabolic differences (Figure S4), particularly demonstrating tight clustering of fermented samples in negative ion mode. Model validation showed excellent fit parameters (Q^2^ = 0.991/0.986; R^2^Y = 1.0/0.998, *p* < 0.05) in both ionization modes, confirming reliable detection of fermentation-induced metabolic alterations.

The 6 h in vitro fermented samples were prioritized to investigate based on the functionally empirical findings. A set of VIP > 1, *p* < 0.05, and Fold Chang (FC) > or < 1.5 was used as filtering criteria. By comparing with control, 24 h, 48 h fermented GTE, a total of 55 signature differential compounds were putatively identified in 6 h fermented GTE by using retention time (RT), m/z value, online databases, and published literature. The classification of chemical families, analogous to protein families, is defined based on homologous members sharing conserved structural skeletons, chemical properties, and analogous catabolic processes [37]. Interestingly, structural classification of microbial bio-transformed 55 GTE metabolites delineated four principal categories, comprising 15 catechins, 29 flavonoids, five organic acids, and six other compounds. In the present study, targeted identification of catechins and flavonoids was achieved using authentic standards, while non-targeted characterization of novel metabolites employed MS/MS spectral matching against authentic spectral libraries and published reference spectra, with strict adherence to mass accuracy thresholds for both precursor and fragment ions. Additionally, the corresponding retention times, molecular formulas, tentative identifications, theoretical and observed mass values, mass errors (Δppm), and characteristic MS/MS fragment ion information of the identified compounds are summarized in Table 1. Specially, as displayed in Table 1, a total of nine phenolic acid metabolites (M1–M9) were identified as potential products of flavonoid glycoside metabolites.

### 3.3. Characterization of Characteristic Compounds and Flavonoid Metabolites in GTE

GTE is rich in polyphenols. Integrating fragmentation patterns of polyphenolic compounds is critical for accurately identifying metabolic pathways and chemical transformations during biotransformation. In Table 1, compounds **1**–**23** all produced the characteristic *m*/*z* 125.02 ion of the MS^2^ fragment. Subclass differentiation emerged through additional ions: compounds **1**–**15** (excluding compound **6**) shared *m*/*z* 137.02, confirming catechin derivatives via database alignment, while **28**–**39** (excluding compounds 33, 36) exhibited *m*/*z* 255.02 with auxiliary ions (*m*/*z* 285.03 and 300.02) specific to kaempferol and quercetin glycosides. Intriguingly, while *m*/*z* 125.02-containing components were not universally classified as catechin derivatives, compounds **16**–**23** carrying this ion were specifically designated flavonoids through integrated analysis of molecular formulae, retention time, and structural congruence. Compounds **45**–**55**, devoid of shared characteristic ions, were unambiguously annotated as diverse entities comprising phenolic acids, theanine, esters, and alkaloids through database interrogation. Notably, compounds **5** and **11** exhibited characteristic catechin-derived secondary fragments during database interrogation, yet their provisional assignment as EGC and EC structural analogs was necessitated by the absence of mass-matched reference catechin structures.

This study conducted qualitative analysis of key flavonoids in GTE samples, concurrently detecting nine green tea flavonoid metabolites. Among these, four catechins, seven flavonoids, and nine phenolic acid metabolites were confirmed by comparison with authentic standards; the remainder underwent preliminary structural characterization by matching characteristic fragment ions to mass spectral databases. Integrating prior catechin metabolism data [27], we further deduced the metabolic pathways of catechins and flavonoids in green tea extract. Taking deprotonated epicatechin (EC) as an example (Figure S5), it undergoes cleavage of its 2-phenylbenzopyran core, generating a NLF from the A-ring via CO_2_ elimination (−44 Da) to form cyclopentadienylpyran (*m*/*z* 245.0793). Simultaneously, cleavage at the C2 position of the BC-ring produces catechol (*m*/*z* 109.0283) and a benzopyran structure (*m*/*z* 179.0331). The conjugated diene system inherent to the benzopyran structure facilitates Retro–Diels–Alder (RDA)-driven fragmentation, yielding DPI such as *m*/*z* 125.0232, *m*/*z* 137.0230, and *m*/*z* 151.0384, which are hallmarks of catechin derivatives. Evidently, a series of catechin analogs, including ECG, EGC, EGCG, along with their conjugated derivatives, exhibited MS/MS profiles analogous to EC. This consistency underscores that RDA reactivity is conserved across catechin congeners sharing the core 2-phenylbenzopyran scaffold. In contrast to EC monomer fragmentation, ECG and EGCG primarily undergo initial cleavage of the C-ring C3-O bond, losing gallic acid (*m*/*z* 109.0283) to form EC/EGC monomers. EGC, however, proceeds via neutral losses (-H_2_O, −18 Da; -CO_2_, −44 Da) followed by RDA-dominated fragmentation. The detailed spectra and cleavage pathways of ECG, EGC and EGCG are displayed in Figures S6–S8, respectively. Based on catechin fragmentation patterns, we propose analogous metabolic pathways for green tea flavonoids. Rutin, after sequential loss of rhamnose and glucose, undergoes oxidation (deprotonation) of the quercetin monomer. Its C-ring then fragments via neutral losses (-CO, −28 Da; -CO_2_, −44 Da), yielding characteristic ions at *m*/*z* 271.0217 and 255.0273. Subsequent RDA cleavage of the B-ring produces ions at m/z 107.0127 and 151.0019 (Figure S9). Quercetin further generates *m*/*z* 243.0269 through consecutive -CO losses, a pattern also observed in kaempferol-3-O-glucoside (astragalin) (Figure S10). Characteristic ions for hyperoside, isoquercitrin, quercetin, taxifolin, nictoflorin and kaempferol in GTE are shown in Figure S11.

### 3.4. Metabolic Fates of Characteristic Flavonoids in GTE by Human Gut Microbiota

The principal constituents of GTE are catechins and flavonoids that share the core C6-C3-C6 skeleton, which underwent analogous microbial modifications in human fecal fermentation, yielding diverse phenolic acid metabolites. In this study, the flavonoids in GTE were extensively metabolized, and were shown the time-dependent dynamic changes in the relative concentrations of diverse phenolic acid metabolites, which are shown in Table 2. The relative amount of quercetin-3-O-galactoside (hyperoside), rutin and isoquercitrin were significantly decreased by 3 h and 6 h of in vitro fermentation, and kaempferol-3-O-rutinoside (nictoflorin) and kaempferol-3-O-glucoside (astragalin) were significantly declined by 3 h of in vitro fermentation. And they were no longer detectable at 12 h, 24 h and 48 h fermentation. In addition, kaempferol-3-O-rutinoside (nictoflorin) and kaempferol-3-O-glucoside (astragalin) were significantly reduced by 3 h in vitro fermentation, and were not detected by 6 h, 12 h, 24 h and 48 h of fermentation. These results indicate that obvious biotransformation occurs during in vitro fermentation. Furthermore, quercetin and dihydroquercetin (taxifolin) were elevated by 3 h or 6 h of fermentation, and then were gradually decreased by further fermentation. Interestingly, an array of phenolic acid metabolites, including 3-(3,4-dihydroxyphenyl) propionic acid, 3-(4-hydroxyphenyl) propionic acid, 3-phenylpropionic acid, 3,4-dihydroxyphenylacetic acid, *p*-hydroxyphenylacetic acid, *m*-hydroxyphenylacetic acid, protocatechuic acid, *p*-hydroxybenzoic acid and phenylacetic acid, were all newly generated by in vitro fermentation compared to unfermented samples.

**Figure 3:**
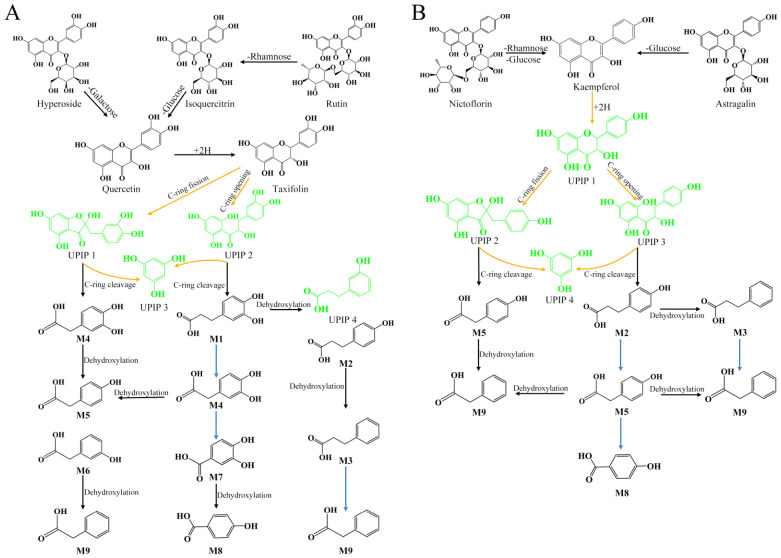
The metabolic pathways of quercetin flavonoid glycosides (**A**) and kaempferol flavonoid glycosides (**B**) in GTE during in vitro fermentation by human gut microbiota. Detected flavonoids or metabolites are shown in black, while putative undetected intermediate products are highlighted in green.

We further investigate how these metabolites generated during in vitro fermentation. As illustrated in Figure 3A,B, rutin hydrolysis initially cleaved *α*-L-rhamnoside to yield quercetin-3-O-glucoside (isoquercitrin), followed by *β*-D-glucosidase-mediated aglycone liberation. Parallel hyperoside deglycosylation via *β*-D-galactoside loss explained the concurrent depletion of these glycosides at 3 h and subsequent quercetin accumulation, maintaining stability until 12 h. Dihydroquercetin (taxifolin), exhibiting synchronous elevation at 6 h consistent with flavonoid glycoside catabolism, underwent complete microbial degradation beyond 24 h, becoming undetectable by 48 h of fermentation. Kaempferol glycosides undergo similar metabolic processes. Kaempferol-3-O-rutinoside (nicotiflorin) and astragalin are hydrolyzed to release rutinoside (rhamnose + glucose) and *β*-D-glucoside, respectively, resulting in the formation of kaempferol. In fact, astragalin showed a marked decrease at 3 h of fermentation, and both nicotiflorin and astragalin were undetectable after 6 h of fermentation. Correspondingly, kaempferol reached its highest concentration at 6 h, indicating that both glycosides were almost completely degraded into kaempferol by that time point. Notably, the dihydro-reduced analog of kaempferol, dihydrokaempferol (aromadendrin), was not detected in this study.

In Figure 3A, we propose two distinct colonic microbial transformation pathways for taxifolin, attributable to instability following C2-C3 of C-ring double bond reduction [38,39]: (1) C-ring cleavage yielding phenylpropionic acid derivatives, and (2) C-ring fission forming cyclopentane-based phenylacetic acid derivatives. Both pathways may share phloroglucinol (UPIP 3) as a common intermediate derived from C-ring cleavage and A-ring modification. In the phenylpropionic acid pathway (C-ring cleavage), the intact B-ring forms 3-(3,4-dihydroxyphenyl) propionic acid (M1). While chalcone intermediates remain undetectable, the resultant 3-(3,4-dihydroxyphenyl) propionic acid undergoes sequential catabolism, including dehydroxylation generated 3-(4-hydroxyphenyl) propionic acid (M2) and undetected 3-(3-hydroxyphenyl) propionic acid (UPIP4)—a putative transient intermediate potentially metabolized to 3-phenylpropionic acid (M3) and phenylacetic acid (M9). Concurrently, M1 underwent side-chain shortening to form 3,4-dihydroxyphenylacetic acid (M4), ultimately forming protocatechuic acid (M7) and dehydroxylated *p*-hydroxybenzoic acid (M8). Alternatively, *m*-hydroxyphenylacetic acid (M6) possibly originated from M4 dehydroxylation or 3-(3-hydroxyphenyl) propionic acid (not detected) metabolism.

In the phenylacetic acid pathway, taxifolin undergoes C-ring fission to a cyclopentane ring, yielding the transient intermediate alphitonin conjugated with the B-ring. Subsequent cleavage of alphitonin’s cyclopentane ring generates an acetic acid side chain on the B-ring, producing metabolite M4. Sequential dehydroxylation of M4 forms *p*-hydroxyphenylacetic acid (M5), M6, and M9. Kaempferol metabolism similarly proceeds via a putative aromadendrin intermediate, undergoing equivalent ring cleavage through both C-ring opening and cyclopentane-forming fission pathways, with phenylacetic acid (M9) as the final colonic metabolite (Figure 3B). Characteristic MS/MS ions of M1-M9 are shown in Figure S12. Notably, Table 2 data reveal post-24 h significant accumulation of flavonoid glycoside metabolites M1, M3, M6, and M9 alongside markedly decreased quercetin and kaempferol levels, demonstrating initial-phase flavonoid glycoside hydrolysis preceding later-stage C-ring metabolism. Collectively, GTE flavonoids undergo human gut microbiota-driven biotransformation through sequential hydrolytic, redox, C-ring opening, C-ring cleavage, dehydroxylation and decarboxylation reactions, culminating in hydroxylated/phenylacetic acid derivatives.

### 3.5. Correlation Analysis of Flavonoids with Antioxidant and Enzyme Inhibitory Activity

**Figure 4:**
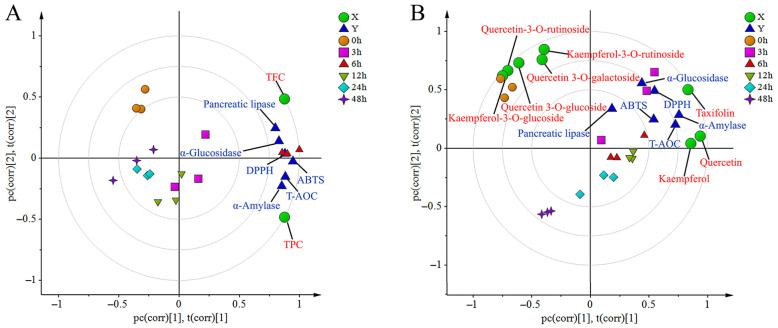
PLS biplot illustrates the correlations between total polyphenols/flavonoids and bioactivities. *X*-axis indicates total polyphenols/flavonoids, *Y*-axis shows six bioactivity indicators (**A**); and between eight metabolites of GTE and six bioactivity indicators. *X*-axis shows eight flavonoids (**B**) at different in vitro fermentation time points (*n* = 3).

Partial least squares (PLS) modeling establishes correlations between GTE bioactivity and flavonoid/polyphenol composition across different fermentation time points (0–48 h). Figure 4A reveals temporal clustering results, and showed PC2 separated 0/3/6 h from 12/24/48 h, while PC1 isolated 3 h and 6 h. Samples at 6 h clustered close to antioxidant (DPPH, ABTS, T-AOC) and enzyme (*α*-glucosidase/amylase, pancreatic lipase) inhibition metrics. TFC changes correlated strongly with pancreatic lipase/*α*-glucosidase inhibition and DPPH, whereas total polyphenols linked closely to ABTS, T-AOC and *α*-amylase inhibition. Figure 4B demonstrates flavonoid-specific associations with some bioactivity indicators. During 0–3 h fermentation, flavonoid glycosides (rutin, hyperoside, isoquercitrin, nictoflorin, astragalin) exhibited negative bioactivity correlations. Conversely, aglycones (taxifolin, quercetin, kaempferol) showed positive associations with bioactivity from 3 to 12 h fermentation, then declined over time. This indicates microbial hydrolysis of glycosides liberates bioactive aglycones, and 6 h fermentation represents the critical phase for enhanced bioactivity via flavonoid biotransformation.

### 3.6. Gut Microbiota Structural–Functional Remodeling Mediated by GTE In Vitro Fermentation

The polyphenol–microbiota crosstalk during in vitro fermentation drives dynamic microbial restructuring, revealing green tea’s prebiotic potential. 16S rRNA profiling of 0, 6, 24 and 48 h fermented samples demonstrated time-dependent microbiota modulation, confirming sufficient sequencing depth (Good’s coverage > 99.8%). Shannon–Wiener and rank–abundance curves are shown in Figure S13. *α*-Diversity metrics showed 48 h fermentation significantly increased the Chao1 index and Ace index by 49.37% and 48.30%, respectively (*p* < 0.05). The Shannon index accordingly had a 0.23-fold increase. However, the Simpson index decreased by 50.44% (*p* < 0.05), which indicates reducing microbial dominance (Figure S13A–F). Multivariate *β*-diversity analysis revealed time-stratified clustering. PLS-DA and PCoA (Bray–Curtis, PERMANOVA R^2^ = 0.69, *p* = 0.004) revealed marked structural microbiota divergence between 0 h and fermented groups (Figure S13G,H). Non-metric multidimensional scaling (NMDS) ordination (stress = 0.02) further validated temporal progression of microbial reorganization, with 48 h fermentation communities occupying distinct phylospace coordinates (Figure S13I,J). Permutational multivariate analysis of variance (PERMANOVA) showed that 48 h fermentation significantly changed the microbiota community compared to unfermented samples. These coordinated shifts demonstrate GTE fermentation induces the micro-ecological network expansion through the diversity of gut microbiota enrichment, as well as functional guild specialization via gut microbiota interaction with polyphenol metabolites.

**Figure 5:**
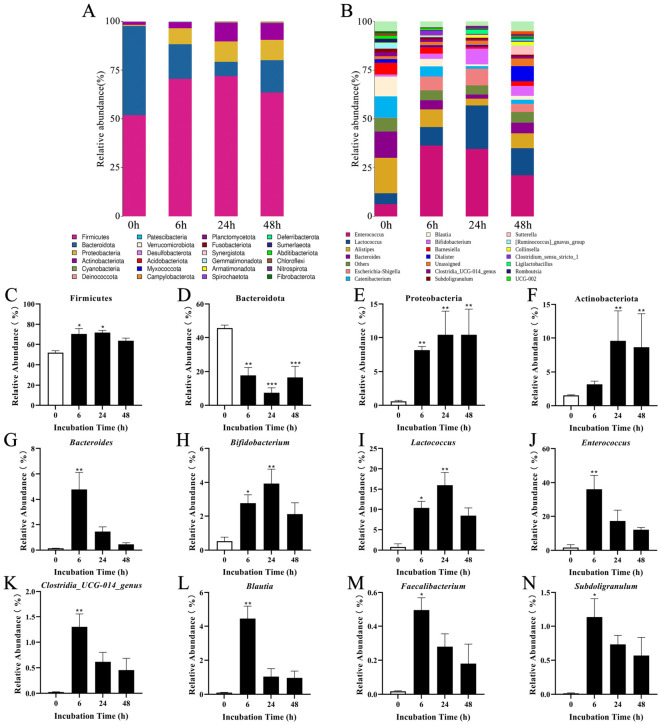
Microbial composition of GTE samples at the phylum and genus levels during in vitro fermentation. Relative abundance at the phylum level (**A**); relative abundance at the genus level (**B**); abundance of dominant phyla: Firmicutes (**C**); Bacteroidota (**D**); Proteobacteria (**E**); Actinobacteriota (**F**); Abundance of selected genera: *Bacteroides* (**G**); *Bifidobacterium* (**H**); *Lactococcus* (**I**); *Enterococcus* (**J**); *Clostridia_UCG-014_genus* (**K**); *Blautia* (**L**); *Faecalibacterium* (**M**); *Subdoligranulum* (**N**). Asterisks indicate significant differences versus the unfermented control: * *p* < 0.05, ** *p* < 0.01, *** *p* < 0.001.

Microbial community restructuring was analyzed across phylogenetic hierarchies. Venn analysis identified 147 conserved OUT/ASVs across the fermentation time course. The total ASV counts demonstrated progressive microbial community expansion (ASVs: 0 h, 601; 6 h, 549; 24 h, 588; 48 h, 758; Figure S14). Phyla-level dynamics revealed Firmicutes, Bacteroidota, Proteobacteria, and Actinobacteriota as core constituents (Figure 5A). However, there was a fermentation-induced significant enrichment in three phyla excluding Bacteroidota (*p* < 0.05). In addition, genus-level profiling revealed a pronounced time-dependent succession of the gut microbiota during in vitro fermentation (0, 6, 24, and 48 h), together with a distinct abundance pattern between dominant taxa and a subset of putative polyphenol-metabolizing specialists (Figure 5B). Temporal phylum shifts initiated at 6 h fermentation, marked by Firmicutes/Proteobacteria proliferation (Figure 5C–F). Notably, the most marked community shift occurred at 6 h, during which the relative abundances of multiple genera increased compared with the unfermented 0 h sample, followed by an overall decrease or redistribution from 24 to 48 h fermentation samples. Specifically, *Bacteroides*, *Enterococcus*, *Clostridia_UCG-014_genus*, *Blautia*, *Faecalibacterium*, and *Subdoligranulum* exhibited an early-enrichment pattern, reaching higher relative abundances at 6 h and subsequently declining (*p* < 0.05; Figure 5G,J,K–N). In contrast, *Bifidobacterium* and *Lactococcus* showed delayed enrichment, with higher relative abundances at 24 h than at 6 h fermentation (*p* < 0.05; Figure 5H,I). Collectively, these results indicate that microbial responses were strongest at the early stage of fermentation and then transitioned toward a rebalanced community structure during the mid-to-late fermentation process. Overall, the observed shifts in microbial composition suggest that GTE exerts selective modulatory effects on the gut microbiota, characterized by stage-specific enrichment of fermentative taxa, bidirectional regulation of flavonoid-metabolizing specialists, and stimulation of selected beneficial bacteria. This coordinated restructuring of the microbial community is consistent with the observed bioactivity profile, supporting the notion that polyphenol-driven ecological selection may underpin the prebiotic potential of green tea.

## 4. Discussion

The health-promoting potential of tea polyphenols, particularly those in green tea, has gained substantial scientific interest due to their multifaceted biological properties. Beyond their well-documented antioxidant capacity, tea polyphenols demonstrate antimicrobial efficacy, metabolic regulatory functions, and protective effects against cardiovascular diseases [10,40,41]. Notwithstanding pharmacokinetic analyses reveal significant bioavailability limitations of tea polyphenols. Additionally, the suboptimal absorption efficiency of tea polyphenols across gastrointestinal segments substantially restricts their systemic bioactivity [42,43]. Current evidence suggests that microbial biotransformation serves as the principal modulator of green tea’s metabolic processing and subsequent bioactivity [44]. Unfortunately, significant knowledge gaps persist regarding the precise microbial transformation mechanisms, strain-specific transformed metabolites, and polyphenol–microbe interactions, particularly considering the differences between in vitro and in vivo metabolism. Future studies employing in vivo animal models are warranted to more precisely characterize the metabolites of tea polyphenols along the gastrointestinal tract, especially in the colon.

The gut microbiota-mediated metabolism of green tea polyphenols has been reported in previous studies. Catechins, particularly EGCG, are primary green tea polyphenols. Liu et al. reported that EGCG supplementation significantly stimulated human gut microbiota, increasing beneficial *Bacteroides*, *Christensenellaceae*, and *Bifidobacterium* while suppressing pathogenic *Fusobacterium varium*. Alongside sequential EGCG transformations via ester hydrolysis, C-ring opening and A-ring fission were observed [26]. Choi et al. studied co-cultured green tea polyphenols with 37 human gut microbiota strains spanning major phyla, and identified *Adlercreutzia*, *Eggerthella*, and *Lactiplantibacillus plantarum* KACC11451 as drivers of catechin C-ring cleavage. *L. plantarum* additionally converted kaempferol-galactoside and quercetin-glucoside to aglycones, which contributed to the enhancement of antioxidant activity [44]. Farag et al. demonstrated microbial biotransformation pathways during in vitro incubation with N-nitrosamines and reported the first evidence of quinone formation from (epi)catechins, quercetin, and kaempferol—a process mitigated by gut microbiota [45]. Further analysis revealed procyanidin B dimer cleavage yields phenylvalerolactones and hydroxylated phenylacetic acids, while flavonoids undergo aglycone reduction to flavonols, C-ring fission to hydroxydihydrochalcones, and ultimately hydroxylated benzoic acid derivatives. Our findings further evidenced this human gut microbiota-mediated biotransfermation of green tea polyphenols.

In fact, flavonoid glycosides and flavonols represent significant dietary polyphenols beyond catechins, whose microbial biotransformation similarly improves gut homeostasis and microbiota composition. Xie et al. identified 49 polyphenols and metabolites (e.g., quercetin, vanillin, *p*-hydroxybenzoic acid) from mung bean coats during in vitro digestion and colonic fermentation, demonstrating colonic microbiota as the primary site for polyphenol liberation and conversion with enhanced antioxidant capacity. Phenolic acid metabolites further synergized with dietary fiber to modulate probiotics (*Lactococcus*, *Bacteroides*) and stimulate short-chain fatty acids (SCFAs) production [46]. Our in vitro fermentation study assessed GTE biotransformation effects on antioxidant activity and enzyme (*α*-glucosidase, *α*-amylase, lipase) inhibition. Chemical analyses revealed significantly enhanced antioxidant activity at 3–6 h versus unfermented GTE (0 h), despite lower efficacy than positive controls. Fermentation supernatants exhibited stronger *α*-glucosidase/amylase inhibition at 6 h, following a similar biphasic pattern (Figure 1). These findings indicate that GTP metabolites, particularly 3′,4′-dihydroxylated derivatives from GTC C-ring cleavage [27,35], enhance bioactivity. Recent evidence confirms flavonols (e.g., quercetin) with C3′-hydroxylated B-rings exert potent *α*-glucosidase/amylase inhibition [27,47,48], supporting fermented green tea (3–6 h) as a potential anti-diabetic supplement. Additionally, flavonoid glycoside deglycosylation (3–6 h) liberating aglycones contributes to elevated antioxidant activity, with declining total flavonoid content post-6 h suggesting progressive conversion [38,44]. Thus, we propose 6 h as an optimal window for maximal in vivo biotransformation efficacy of green tea polyphenols.

Our group previously characterized four catechins, eight flavonoids, and nine flavonoid metabolites during green tea catechins fermentation by HGM [27]. Catechins underwent C-ring opening and A-ring fission to form diphenylpropanols, phenylvalerolactones, and phenylvaleric acids (e.g., 1-(3′-hydroxyphenyl)-3-(2″,4″,6″-trihydroxyphenyl)-propan-2-ol, pyrogallol) [27,44]. Unlike catechins, most native flavonoids exist as C-/O-glycosides, requiring microbial conversion to bioavailable phenolic acids for absorption [49]. As shown in Table 2, rutin, quercetin 3-O-glucoside, and astragalin decreased significantly by 3 h fermentation, then gradually became undetectable by 6–12 h fermentation. At the same time, quercetin/kaempferol was found to increase coincidingly. This indicates that microbial *β*-glucosidase/*α*-L-rhamnosidase-mediated deglycosylation occurs, which reaches its peak at 6 h and is completed by 12 h of fermentation [50,51]. Aglycones were reduced to create dihydroflavonols (taxifolin, aromadendrin) via two pathways: C-ring opening yielding phenylpropionic acids or C-ring fission forming cyclopentane intermediates en route to phenylacetic acids. Both pathways generate phloroglucinol and 3,4-dihydroxyphenylacetic acid, culminating in phenylacetic acid. The concentration of quercetin, kaempferol, and taxifolin peaked at 6 h, but were largely metabolized by 24 h fermentation. Concurrently, phenolic acids (e.g., 3-(3,4-dihydroxyphenyl) propionic acid, 3,4-dihydroxyphenylacetic acid) accumulated post-24 h via dehydroxylation and *β*-oxidation-like side-chain shortening [37,38,52]. While phenylacetic acids are established anaerobic metabolites, in vivo hepatic modifications may yield methylated/acetylated derivatives (e.g., 3-methoxy-4-hydroxyphenylacetic acid, phenylacetylglycine) [38]. Additionally, correlation analysis found enhanced bioactivity at 6 h fermentation may primarily derive from catechin metabolites (e.g., EGCG derivatives), with flavonoid biotransformation critically augmenting activity post-3 h fermentation. Thus, GTE flavonoids are deglycosylated by microbial hydrolases, reduced to form unstable dihydroflavonols (disrupting C-ring conjugation), and cleaved via C-ring opening/fission pathways, ultimately generating phenylacetic acid derivatives through sequential dehydroxylation and side-chain shortening.

GTE polyphenols remain largely unaltered prior to colonic arrival, where gut microbiota, particularly anaerobes, determine their biotransformation fate [38,53]. Emerging research delineates a bidirectional interplay between green tea polyphenols and gut microbial consortia, where microbial enzymatic transformations dictate polyphenol bioactivation while polyphenol metabolites reciprocally modulate microbial ecology [24,54]. Actually, this conserved biotransformation cascade is mediated by gut microbial enzymatic activity, where individual taxa express strain-specific catalytic repertoires that drive divergent metabolic outcomes [37,39]. This in vitro fermentation demonstrated significantly enhanced *α*- and *β*-diversity compared to unfermented controls, indicating improved microbial richness, structural reorganization, and functional remodeling. Crucially, microbial abundance shifts temporally aligned with peak bioactivity at 6 h, implying GTE polyphenol metabolites drive microbial dynamics. The eight bacterial genera identified in this study may participate in a sequential and complementary manner, consistent with a “division-of-labor” model characteristic of gut microbial metabolism [55]. The initial and potentially rate-limiting step involves deglycosylation, which expands the pool of aglycones (specifically quercetin and kaempferol) available for subsequent metabolic transformations. Here, *Bacteroides* is plausibly involved in removing terminal rhamnose from rutinosides, whereas *Bifidobacterium* is a strong candidate for hydrolyzing glucose-conjugated flavonoids via *β*-glucosidase, thereby releasing aglycones from isoquercitrin-/astragalin-type substrates [50,56,57]. *Lactococcus* and *Enterococcus*, both frequently associated with glycosidase activities in fermentative environments, may further expand the deglycosylation capacity of the consortium, accelerating early-stage conversion of glycosides to aglycones [58,59]. Following deglycosylation, the +2H reduction and subsequent C-ring opening/fission/cleavage likely reflect specialized anaerobic activities that are difficult to assign at the genus level based on 16S RNA data alone. Nevertheless, the pronounced dynamics of *Clostridia_UCG-014_genus* suggest a potential link to downstream reductive aromatic transformations [60]. In contrast, *Blautia*, *Faecalibacterium*, and *Subdoligranulum* are more plausibly positioned as secondary consumers, benefiting from cross-feeding on sugars released during deglycosylation and on small phenolic intermediates, thereby contributing to community rebalancing at later fermentation stages [60,61]. The current in vitro evidence on GTE polyphenol metabolites is limited by the use of 16S rRNA sequencing, which only allows genus-level identification. Since polyphenol biotransformation is frequently species- or strain-specific, shotgun metagenomics would be required in future studies to pinpoint the functional genes involved. Overall, catechin/flavonoid metabolism and selective microbial enrichment appear mutually reinforcing, jointly shaping polyphenol transformation and potentially contributing to intestinal health.

## 5. Conclusions

In conclusion, our findings demonstrate a dynamic interaction between GTE and HGM during 48 h of in vitro anaerobic fermentation. Initial assessments of antioxidant activity, *α*-glucosidase, *α*-amylase, and pancreatic lipase inhibition revealed a time-dependent biphasic pattern in GTE bioactivity, peaking at 6 h of fermentation. And this optimal time point for polyphenol bioactivity was found positively correlated to total polyphenol and flavonoid content. Subsequent UHPLC-Q-Orbitrap-MS/MS-based untargeted metabolomics characterized 55 signature compounds in GTE and identified nine phenolic acid metabolites derived from GTE flavonoids, suggesting two potential metabolic pathways culminating in phenylacetic acid via ring fission of the flavonol C-ring. Furthermore, 16S rRNA sequencing data revealed significant restructuring of microbial diversity and composition induced by GTE polyphenol metabolites, with notably increased abundance of *Bacteroides*, *Bifidobacterium*, *Lactococcus* and *Enterococcus* post-6 h fermentation, when coinciding with peak bioactivity, implying their potential involvement in further flavonoid metabolism. This microbiota-mediated biotransformation of GTE provides mechanistic insight into green tea’s health benefits what may happen in human intestines. Further investigations are needed to identify specific species of microbiota to produce enzymes catalyzing tea polyphenols, and to screen potential polyphenols metabolites to execute function in the human body when drinking tea.

## Figures and Tables

**Table 1:** Characteristic metabolites of fermented GTE identified from UHPLC-Q-Orbitrap-MS/MS data by human gut microbiota in vitro fermentation.

NO.	Tentative Identification	Formula	Theoretical Mass [M-H]	Detected Mass [M-H]	Error (ppm)	RT [min]	FC (Log_2_(C/A))	MS^2^ Fragment
1	EC ^a^	C_15_H_14_O_6_	289.07066	289.07068	0.05	9.58	−2.11	125.0232, 137.0231, 151.0388, 179.0329, 245.0793
2	EGC ^a^	C_15_H_14_O_7_	305.06558	305.06577	0.63	10.32	−1.65	125.0230, 137.0227, 167.0330, 179.0330, 219.0643
3	ECG ^a^	C_22_H_18_O_10_	441.08162	441.08127	−0.80	11.39	−4.13	125.0232, 137.0229, 151.0385, 169.0124, 289.0683
4	EGCG ^a^	C_22_H_18_O_11_	457.07654	457.07712	1.27	10.86	−2.98	125.0231, 137.0230, 169.0123, 243.0640, 305.0623
5	EGC derivative	C_19_H_18_O_6_	341.10196	341.10184	−0.37	9.35	−1.09	125.0231, 137.0228, 167.0325, 179.0328, 305.0630
6	6-Carboxyl-(-)-gallocatechin	C_16_H_14_O_9_	349.05541	349.05588	1.35	8.16	−2.45	78.9582, 97.0286, 125.0343, 167.4214, 223.0344
7	8-C-ascorbyl (-)-epigallocatechin	C_21_H_20_O_13_	479.08202	479.08194	−0.16	10.63	−1.41	125.0232, 137.0227, 151.0023, 169.0125, 178.9966
8	3-O-Methyl-epicatechin	C_15_H_12_O_7_	303.04993	303.04999	0.20	9.10	−1.80	125.0231, 137.0228, 151.0381, 165.0173, 229.0485
9	EC 3-(4-O-Methy)-gallate	C_23_H_20_O_10_	455.09727	455.09653	−1.63	10.41	−1.59	125.0231, 137.0232, 169.0129
10	(-)-Epiafzelechin 3-O-gallate	C_22_H_18_O_9_	425.08671	425.08685	0.33	12.21	−3.75	125.0231, 137.0228, 169.0123, 229.0842, 273.0738
11	Catechin derivative	C_19_H_18_O_7_	357.09688	357.09650	−1.06	9.77	−1.49	125.0231, 137.0229, 151.0382, 169.0954, 289.0681
12	EGC 3-(3-OMe)-gallate	C_23_H_20_O_11_	471.09219	471.09293	1.58	11.14	−1.17	125.0232, 137.0226, 168.0045, 183.0276, 305.0626
13	(+)-Catechin (4*α*-8)-(-)-epigallocatechin 3-O-gallate	C_37_H_30_O_17_	745.13993	745.13745	−3.32	10.04	−4.59	125.0230, 137.0232, 151.0387, 289.0685, 423.0684
14	Epigallocatechin-(4*β*→8)-catechin	C_30_H_26_O_13_	593.12897	593.12854	−0.72	9.32	−1.04	125.0230, 137.0228, 179.0326, 255.0278, 305.0628
15	(-)-Gallocatechin 3,5-di-O-gallate	C_29_H_22_O_15_	609.08750	609.08777	0.45	8.76	−1.88	125.0230, 137.0231, 165.0171, 255.0268, 305.0638
16	Procyanidin B2	C_30_H_26_O_12_	577.13405	577.13281	−2.15	10.02	−2.26	125.0231, 137.0228, 169.0961, 255.0273, 289.0684
17	Procyanidin B2 3′-gallate	C_37_H_30_O_16_	729.14501	729.14418	−1.14	10.58	−4.60	125.0230, 137.0228, 169.0121, 289.0689, 407.0735
18	Eriodictyol	C_15_H_12_O_6_	287.05501	287.05563	2.14	13.11	1.40	125.0230, 151.0024, 177.0539, 259.0577
19	Galloylquercitrin	C_28_H_24_O_15_	599.10315	599.10345	0.51	9.91	1.79	125.0231, 151.0023, 169.0123, 255.0268, 284.0291
20	Cyanidin 3-O-sambubioside chloride	C_26_H_29_ClO_15_	615.11112	615.11121	0.14	10.44	−1.88	125.0231, 137.0230, 161.0382, 269.0424, 287.0542
21	Myricetin 3-O-galactoside	C_21_H_20_O_13_	479.08202	479.08286	1.76	10.80	−1.41	125.0232, 271.0216, 287.0167, 316.086, 317.0266
22	Dihydromyricetin	C_15_H_12_O_8_	319.04484	319.04565	2.53	10.39	1.01	125.0232, 151.0021, 165.0159, 193.0119, 301.0312
23	Prodelphinidin B	C_30_H_26_O_14_	609.12388	609.12439	0.83	11.86	−3.74	125.0230, 137.0231, 177.0131, 255.0268, 305.0638
24	Schaftoside	C_26_H_28_O_14_	563.13953	563.14032	1.40	10.21	−3.86	297.0732, 353.0623, 383.0722, 443.0927, 473.1042
25	Isoschaftoside	C_26_H_28_O_14_	563.13953	563.13927	−0.46	10.68	−1.02	297.0735, 353.0627, 383.0728, 443.0956, 503.1152
26	Naringenin-7-O-*β*-primeveroside	C_26_H_30_O_14_	565.15518	565.15564	0.81	9.35	−3.14	227.0594, 228.0670, 246.0228, 285.1366, 303.0927
27	Poncirin	C_28_H_34_O_14_	593.18648	593.18646	−0.04	11.40	−1.68	227.0325, 255.0270, 284.0294, 285.0372, 593.1433
28	Kaempferol-3-O-galactoside-7-O-rhamnoside	C_27_H_30_O_15_	593.15010	593.14911	−1.66	11.55	−1.78	227.0326, 229.0479, 255.0270, 284.0291, 285.0375
29	Kaempferol-3-O-rutinoside (Nictoflorin)	C_27_H_30_O_15_	593.15010	593.15112	1.73	13.39	−1.04	227.0326, 255.0268, 284.0295, 285.0373
30	Kaempferol-3-O-glucoside (Astragalin) ^a^	C_21_H_20_O_11_	447.09219	447.09213	−0.13	11.71	−4.99	227.0324, 255.0268, 284.0293, 285.0374, 447.0879
31	Kaempferol 3-(6′-rhamnosylsophoroside)	C_33_H_40_O_20_	755.20292	755.20428	1.80	11.09	−1.12	227.0326, 255.0270, 285.0373, 755.1931
32	Kaempferol-3-O-glucosyl-rhamnosyl-glucoside	C_33_H_40_O_20_	755.20292	755.20270	−0.29	11.24	−1.11	227.0324, 255.0269, 284.0294, 285.0372
33	Kaempferol ^a^	C_15_H_10_O_6_	285.03936	285.04047	3.89	8.34	5.89	227.0592, 228.0669, 285.1365
34	Quercetin 3-rhamnosyl-(1→2)-rhamnosyl-(1→6)-glucoside	C_33_H_40_O_20_	755.20292	755.20270	−0.29	10.89	−2.43	255.0270, 271.0211, 300.0236, 301.0319, 755.1918
35	Quercetin-3-O-galactosyl-rhamnosyl-glucoside	C_33_H_40_O_21_	771.19783	771.19965	2.35	10.59	−1.05	255.0273, 271.0219, 300.0237, 301.0310
36	Quercetin 3-O-arabinoside	C_20_H_18_O_11_	433.11292	433.11298	0.13	7.76	1.44	125.0232, 169.0123, 190.9941, 365.0447
37	Quercetin-3-O-galactoside (Hyperoside) ^a^	C_21_H_20_O_12_	463.08710	463.08615	−2.06	11.29	−3.81	255.0266, 271.0215, 300.0238, 301.0315
38	Quercetin 3-O-glucoside (Isoquercitrin) ^a^	C_21_H_20_O_12_	463.08710	463.08728	0.38	11.56	−4.85	125.0231, 137.0229, 255.0268, 271.0218, 300.0241
39	Quercetin-3-O-rutinoside (Rutin) ^a^	C_27_H_30_O_16_	609.14501	609.14398	−1.69	11.21	−4.84	151.0020, 243.0269, 255.0271, 271.0217, 300.0242
40	Dihydroquercetin (Taxifolin) ^a^	C_15_H_12_O_7_	303.04993	303.05093	3.30	10.34	5.40	227.0589, 228.0671, 285.1366
41	Quercetin ^a^	C_15_H_10_O_7_	301.03428	301.03534	3.52	10.45	5.47	107.0127, 151.0019, 178.9964, 273.0395, 301.0320
42	Apigenin	C_15_H_10_O_5_	269.04445	269.04385	−2.23	14.95	2.07	107.0130, 117.0334, 121.0280, 151.0020, 269.0424
43	Apigenin-8-C-glucoside (Vitexin)	C_21_H_20_O_10_	431.09727	431.09723	−0.10	11.20	−1.54	121.0282, 161.0229, 283.0580, 311.0525, 341.0629
44	Vitexin 2″-O-rhamnoside	C_27_H_30_O_14_	577.15518	577.15619	1.75	10.87	−1.52	269.0430, 293.0423, 311.0526, 413.0833
45	Gallic acid ^a^	C_7_H_6_O_5_	169.01315	169.01311	−0.23	7.39	−3.28	79.0182, 81.0339, 97.0285, 125.0231, 169.0123
46	3-O-Galloylquinic acid (Theogallin)	C_14_H_16_O_10_	343.06597	343.06683	2.50	7.18	−1.05	125.0231, 169.0124, 173.0431, 191.0537
47	Quinic acid	C_7_H_12_O_6_	191.05501	191.05489	−0.65	2.07	4.20	85.0288, 87.0080, 93.0338, 173.0437, 191.0543
48	5-O-*p*-Coumaroylquinic acid	C_16_H_18_O_8_	337.09179	337.09195	0.46	9.39	−1.06	119.0489, 163.0382, 191.0539, 173.0437
49	L-theanine	C_7_H_14_N_2_O_3_	173.09207	173.09187	−1.16	10.37	−1.15	93.0336, 130.0859, 173.0435
50	Gallic acid 4-O-(6-galloylglucoside)	C_20_H_20_O_14_	483.07693	483.07675	−0.38	9.03	−3.83	123.0075, 124.0153, 125.0230, 168.0044, 169.0122
51	1,2,6-Trigalloyl glucose	C_27_H_24_O_18_	635.08789	635.08813	0.38	10.14	−2.12	125.0232, 151.0022, 169.0125, 313.0524, 465.0633
52	Methyl gallate	C_8_H_8_O_5_	183.02880	183.02849	−1.69	11.54	1.26	124.0153, 139.0751, 168.0054, 183.0643
53	1-Galloylglucose	C_13_H_16_O_10_	331.06597	331.06531	−2.00	2.47	−1.18	125.0230, 169.0122, 211.0267, 271.0426, 331.0629
54	Caffeine	C_8_H_10_N_4_O_2_	195.08765 [M + H]	195.08818 [M + H]	2.72	5.37	−1.02	110.0699, 125.0694, 138.0645, 195.0853
55	Theobromine	C_7_H_8_N_4_O_2_	179.05635	179.05631	−0.22	9.46	−1.02	90.9971, 134.9864, 179.0556
M1	3-(3,4-dihydroxyphenyl)propionic acid ^a^	C_9_H_10_O_4_	181.04954	181.04913	−2.26	10.19	3.06	59.0132, 109.0283, 121.0281, 137.0591, 181.0486
M2	3-(4-hydroxyphenyl)propionic acid ^a^	C_9_H_10_O_3_	165.05462	165.05424	−2.30	9.76	2.12	119.0489, 121.0646, 147.0435, 165.0534
M3	3-phenylpropionic acid ^a^	C_9_H_10_O_2_	149.05971	149.05923	−3.22	14.58	3.71	59.0132, 94.9930, 104.9760, 121.0287
M4	3,4-dihydroxyphenylacetic acid ^a^	C_8_H_8_O_4_	167.03389	167.03384	−0.30	10.36	4.82	123.0803
M5	*p*-hydroxyphenylacetic acid ^a^	C_8_H_8_O_3_	151.03897	151.03864	−2.18	7.37	4.55	63.0026, 106.9916
M6	*m*-hydroxyphenylacetic acid ^a^	C_8_H_8_O_3_	151.03897	151.03856	−2.72	9.47	2.21	93.0336, 106.9918, 107.0489, 123.0438
M7	protocatechuic acid ^a^	C_7_H_6_O_4_	153.01824	153.01817	−0.46	11.00	2.57	65.0389, 109.0283, 135.0072, 153.0176
M8	*p*-hydroxybenzoic acid ^a^	C_7_H_6_O_3_	137.02332	137.02325	−0.51	10.06	1.31	65.0389, 93.0336, 137.0230
M9	phenylacetic acid ^a^	C_8_H_8_O_2_	135.04406	135.04402	−0.30	11.74	2.93	65.0137, 90.9972, 92.0245, 135.0298

^a^: Confirmed by comparison with commercial standards; RT: retention time; FC: fold change; M: metabolites; C: 6 h fermented group; A: 0 h unfermented group; EC: epicatechin; EGC: epigallocatechin; ECG: epicatechin gallate; EGCG: epigallocatechin gallate.

**Table 2:** The relative contents of flavonoid compounds and metabolites in GTE with human intestinal microbiota at 0, 3, 6, 12, 24, 48 h, respectively (*n* = 3).

Compounds	Concentration (μg/mL)					
	0 h	3 h	6 h	12 h	24 h	48 h
Quercetin-3-O-galactoside (Hyperoside)	1.2036 ± 0.2598	0.6469 ± 0.5537	0.3411 ± 0.0176	-	-	-
Quercetin-3-O-rutinoside (Rutin)	27.4423 ± 6.3522	4.3141 ± 2.9201	0.0785 ± 0.0387	-	-	-
Quercetin 3-O-glucoside (Isoquercitrin)	15.2061 ± 5.3194	4.0093 ± 2.7752	0.5974 ± 0.2443	-	-	-
Quercetin	0.1823 ± 0.1276	11.6690 ± 3.5108	12.1146 ± 2.5429	10.4712 ± 1.5868	8.0580 ± 2.7740	1.5873 ± 0.4034
Dihydroquercetin (Taxifolin)	0.1481 ± 0.0615	0.8978 ± 0.2708	0.7529 ± 0.1014	0.5897 ± 0.0628	0.2653 ± 0.0794	-
Kaempferol-3-O-rutinoside (Nictoflorin)	0.8262 ± 0.1209	0.5975 ± 0.1281	-	-	-	-
Kaempferol-3-O-glucoside (Astragalin)	6.1798 ± 0.6889	0.1975 ± 0.0446	-	-	-	-
Kaempferol	0.0272 ± 0.0032	3.4695 ± 1.8895	5.0495 ± 1.4216	3.1418 ± 1.7821	3.9665 ± 1.3555	0.6394 ± 0.1371
3-(3,4-dihydroxyphenyl)propionic acid	-	0.1388 ± 0.0306	0.1727 ± 0.0737	0.1777 ± 0.0598	2.5523 ± 0.6274	2.8315 ± 0.4968
3-(4-hydroxyphenyl)propionic acid	-	-	-	-	2.8456 ± 0.9110	1.4682 ± 0.4286
3-phenylpropionic acid	-	0.1597 ± 0.0900	0.1439 ± 0.0372	0.1768 ± 0.1003	5.4258 ± 0.7569	6.4184 ± 0.7748
3,4-dihydroxyphenylacetic acid	-	-	0.4596 ± 0.1576	-	1.1588 ± 1.1465	3.5792 ± 1.2679
*p*-hydroxyphenylacetic acid	-	0.2913 ± 0.2595	0.5999 ± 0.1297	0.3545 ± 0.0406	0.9791 ± 0.4473	4.2888 ± 1.5882
*m*-hydroxyphenylacetic acid	-	-	0.1169 ± 0.0446	0.4456 ± 0.1994	2.7995 ± 2.1373	4.7094 ± 2.1667
protocatechuic acid	-	-	-	-	-	4.6293 ± 0.6640
*p*-hydroxybenzoic acid	-	-	-	-	-	0.1066 ± 0.0034
phenylacetic acid	-	0.2078 ± 0.0334	0.2302 ± 0.0772	0.2302 ± 0.0632	3.2249 ± 0.5422	3.3132 ± 0.5616

Note: The concentrations of these metabolites and flavonoids were calculated through the linear curve of the EGCG standard (R^2^ > 0.99).

## Data Availability

The original contributions presented in the study are included in the article/Supplementary Material; further inquiries can be directed to the corresponding authors.

## Supplementary Materials

The following supporting information can be downloaded at: https://www.mdpi.com/article/10.3390/foods15101732/s1, Figure S1: Standard curves for antioxidant and polyphenol quantification. Trolox standard measured at 593 nm (A), gallic acid standard measured at 765 nm (B), and rutin standard measured at 510 nm (C). Figure S2: TICs of GTE samples and unfermented samples, showing comparisons of TIC chromatograms between unfermented GTE samples and QC samples (A): QC samples in negative ion mode (a), QC samples in positive ion mode (b), 0 h samples in negative ion mode (c), 0 h samples in negative ion mode (d), and between fermented GTE samples and QC samples (B): GTE samples in positive ion mode (a), GTE samples in negative ion mode (b), QC samples in positive ion mode (c), QC samples in negative ion mode (d). Figure S3: TIC chromatography of GTE at different in vitro fermentation time. Figure S4: OPLS-DA of UHPLC-Orbitrap-MS/MS data from unfermented 0 h and fermented 6 h GTE samples from negative ion (A) and positive ion modes (B), respectively (*n* = 4), permutation test models for sample data from negative ion (C) and positive ion modes (D) (*n* = 100). Figure S5: The MS/MS spectrometry of EC (A) and its cleavage pathway (B). Figure S6: The MS/MS spectrometry of ECG (A) and its cleavage pathway (B). Figure S7: The MS/MS spectrometry of EGC (A) and its cleavage pathway (B). Figure S8: The MS/MS spectrometry of EGCG (A) and its cleavage pathway (B). Figure S9: The MS/MS spectrometry of rutin (A) and its cleavage pathway (B). Figure S10: The MS/MS spectrometry of astragalin (A) and its cleavage pathway (B). Figure S11: The MS/MS spectrum of hyperoside (A), isoquercitrin (B), quercetin (C), taxifolin (D), nictoflorin (E) and kaempferol (F) in GTE. Figure S12: The MS/MS spectrum of M1 (A), M2 (B), M3 (C), M4 (D), M5 (E), M6 (F), M7 (G), M8 (H) and M9 (I). Figure S13: Microbial community analysis of GTE samples during in vitro fermentation with HGM via 16S rRNA sequencing. Venn diagram of OTUs/ASVs among samples fermented for 0, 6, 24, and 48 h (A); hierarchical clustering of GTE samples across fermentation time points (B); Shannon–Wiener curves of samples fermented for 0, 6, 24, and 48 h (C); rank–abundance curves of samples fermented for 0, 6, 24, and 48 h (D). Figure S14: Effects of various fermentation time (0 h, 6 h, 24 h and 48 h) of GTE on the *α*- and *β*-diversity of the gut microbiota. Observed species (A); Chao1 index (B); ACE index (C); Shannon index (D); Simpson index (E); Goods coverage (F); PLS-DA score plot (G); PCoA plot (H); NMDS plot (I); PERMANOVA (J). Different lowercase letters indicate significant differences (*n* = 3, *p* < 0.05). Table S1: Basic information of volunteers. Table S2: Relative average contents of total antioxidant activity, total polyphenols and total flavonoids in GTE at different fermentation time points (*n* = 3).
