# Supplementary material for: In Vitro Fermentation of Green Tea by Human Gut Microbiota Enhances Bioactivity and Bidirectionally Modulates Polyphenol Metabolites and Gut Microbiota
Source: Foods. 2026 May 14;15(10):1732. doi: 10.3390/foods15101732 (PMC13206288; doi:10.3390/foods15101732)
Supplement: Supplementary file 1 [file foods-15-01732-s001.zip › foods-4286728-supplementary.pdf]

Figure S1. Standard curves for antioxidant and polyphenol quantification. Trolox standard measured at 593 nm (A), gallic acid standard measured at 765 nm (B), and rutin standard measured at 510 nm (C).

Figure S2. TIC chromatograms of GTE unfermented samples, showing comparisons of TIC chromatograms between unfermented GTE samples and QC samples (A): QC samples in negative ion mode (a), QC samples in positive ion mode (b), 0 h samples in negative ion mode (c), 0 h samples in negative ion mode (d); and between fermented GTE samples and QC samples (B): GTE samples in positive ion mode (a), GTE samples in negative ion mode (b), QC samples in positive ion mode (c), QC samples in negative ion mode (d). The QC sample represents a pooled mixture of GTE samples at different fermentation times.

Figure S3. TIC chromatography of GTE at 0h, 3h, 6h, 12h, 24h and 48h in vitro fermentation time, respectively.

Figure S4. OPLS-DA of UHPLC-Orbitrap-MS/MS data from fermented 0 h and 6 h GTE samples. (A) and (B) represent the data analysis under negative ion and positive ion modes, respectively (n = 4), while (C) and (D) illustrate the permutation test models used for sample data analysis under the corresponding ionization modes (n = 100).

Figure S5. The MS^2 spectrometry of EC and its cleavage pathway.

Figure S6. The MS^2 spectrometry of ECG and its cleavage pathway.

Figure S7. The MS^2 spectrometry of EGC and its cleavage pathway.

Figure S8. The MS^2 spectrometry of EGCG and its cleavage pathway.

Figure S9. The MS^2 spectrometry of rutin and its cleavage pathway.

Figure S10. The MS^2 spectrometry of astragalin and its cleavage pathway.

Figure S11. The MS^2 spectrum of (A) Hyperoside, (B) Isoquercitrin, (C) Quercetin, (D) Taxifolin; (E) Nictoflorin and (F) Kaempferol in GTE.

Figure S12. The MS^2 spectrum of (A) M1, (B) M2, (C) M3, (D) M4, (E) M5, (F) M6, (G) M7, (H) M8 and (I) M9.

Figure S13. Effects of GTE fermentation time (0h, 6h, 24h and 48h) on the α- and β-diversity of the gut microbiota. (A) Observed species; (B) Chao1 index; (C) ACE index; (D) Shannon index; (E) Simpson index; (F) Goods coverage; (G) PLS-DA score plot; (H) PCoA plot; (I) NMDS plot; (J) PERMANOVA. Different lowercase letters indicate significant differences (n=3, $p < 0.05$).

Figure S14. Microbial community analysis of GTE samples during in vitro fermentation via 16S rRNA sequencing. (A) Venn diagram of OTUs/ASVs among samples fermented for 0, 6, 24, and 48 h; (B) Hierarchical clustering of GTE samples across fermentation time points; (C) Shannon–Wiener curves of samples fermented for 0, 6, 24, and 48 h; (D) Rank-abundance curves of samples fermented for 0, 6, 24, and 48 h.

Table S1. Basic information of voluntary donors.

Table S2. Relative average contents of total antioxidant activity, total polyphenols and total flavonoids in GTE at different fermentation time points (n=3).

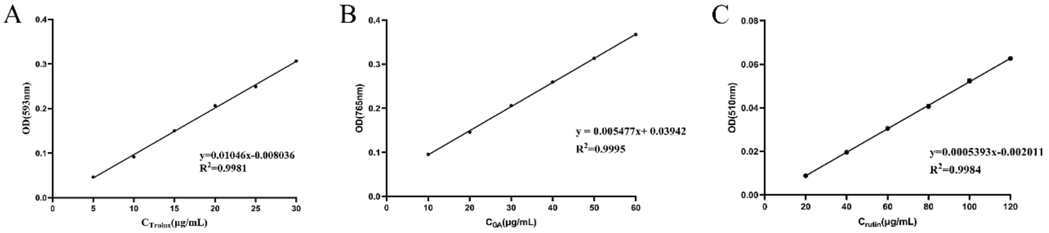

Figure S1. Standard curves for antioxidant and polyphenol quantification. Trolox standard measured at 593 nm (A), gallic acid standard measured at 765 nm (B), and rutin standard measured at 510 nm (C).

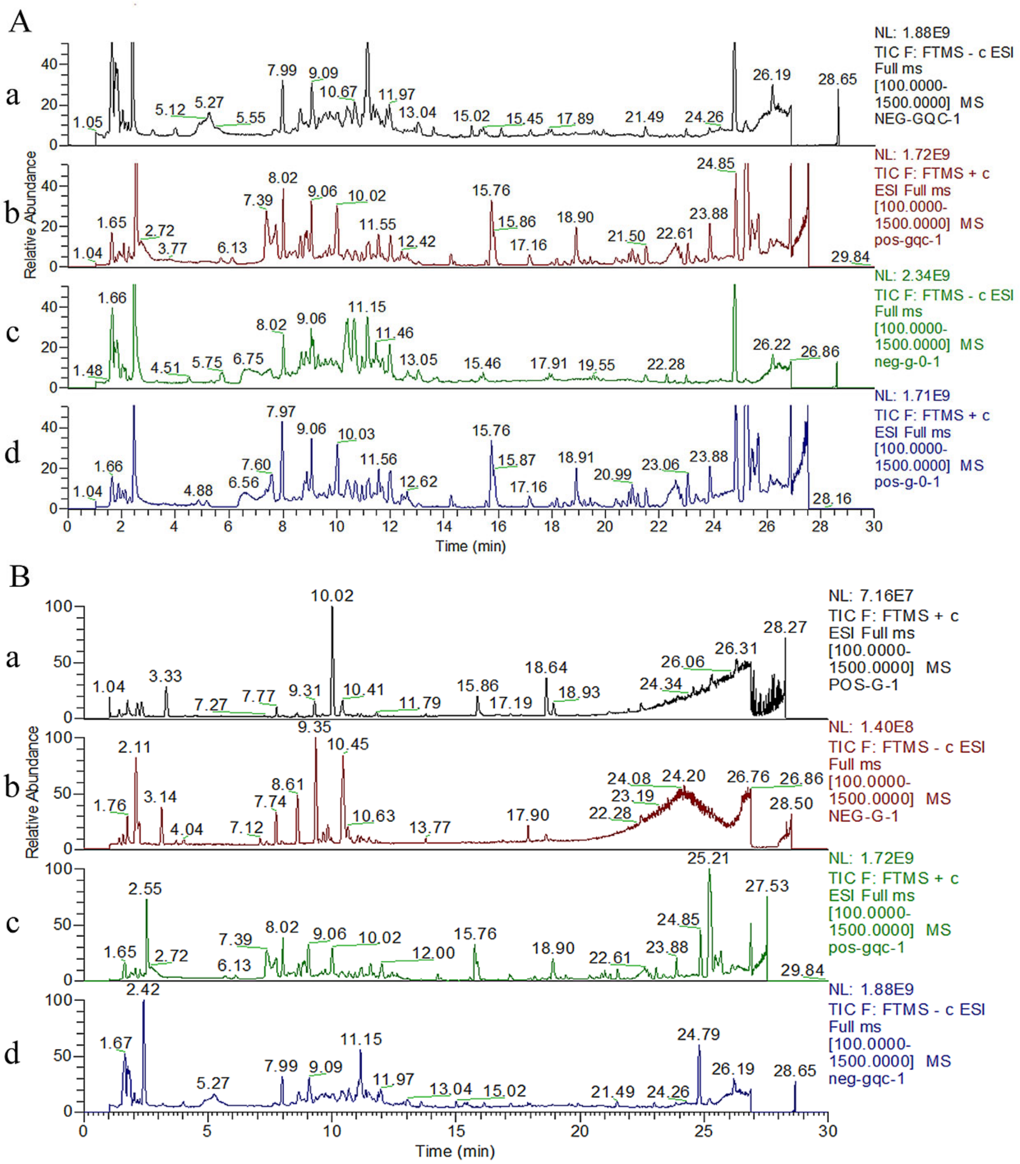

Figure S2. TIC chromatograms of GTE unfermented samples, showing comparisons of TIC chromatograms between unfermented GTE samples and QC samples (A): QC samples in negative ion mode (a), QC samples in positive ion mode (b), 0 h samples in negative ion mode (c), 0 h samples in negative ion mode (d); and between fermented GTE samples and QC samples (B): GTE samples in positive ion mode (a), GTE samples in negative ion mode (b), QC samples in positive ion mode (c), QC samples in negative ion mode (d). The QC sample represents a pooled mixture of GTE samples at different fermentation times.

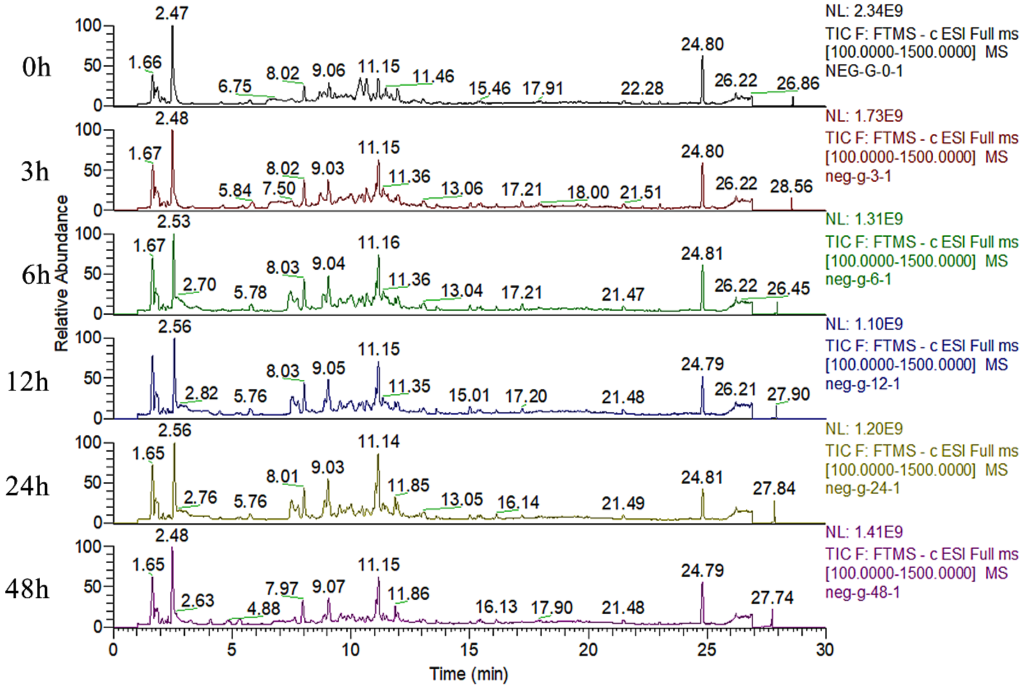

Figure S3. TIC chromatography of GTE at 0h, 3h, 6h, 12h, 24h and 48h in vitro fermentation time, respectively.

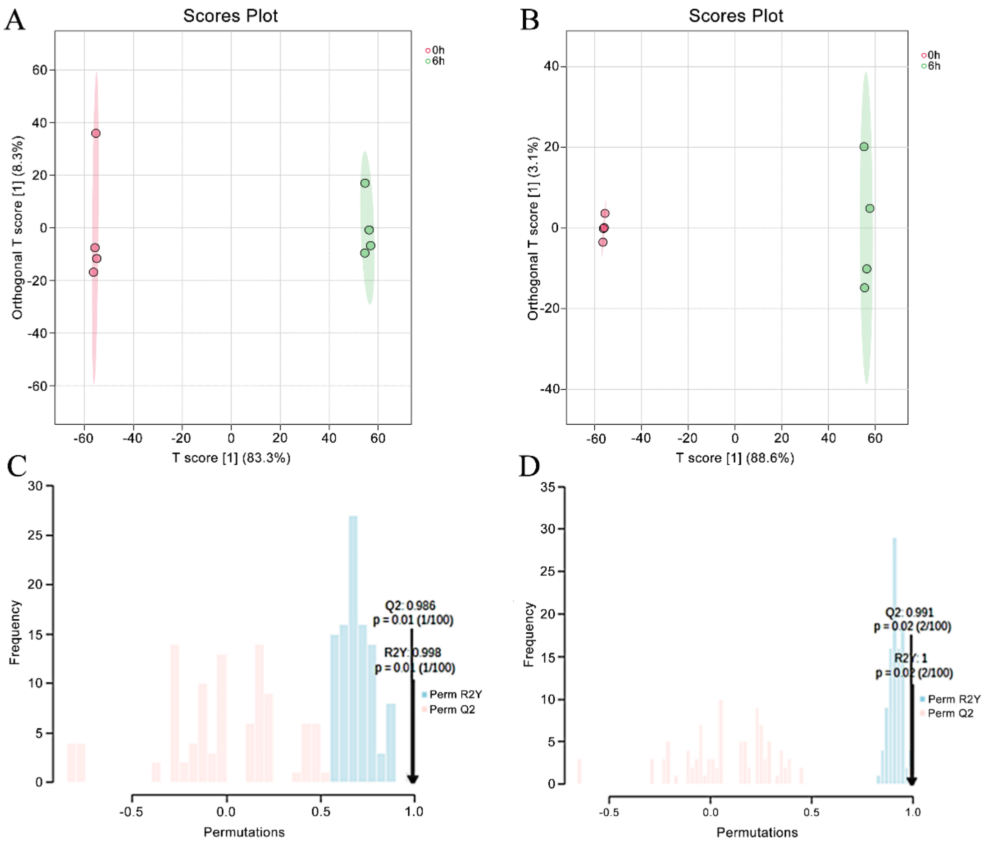

Figure S4. OPLS-DA of UHPLC-Orbitrap-MS/MS data from fermented 0 h and 6 h GTE samples. (A) and (B) represent the data analysis under negative ion and positive ion modes, respectively (n = 4), while (C) and (D) illustrate the permutation test models used for sample data analysis under the corresponding ionization modes (n = 100).

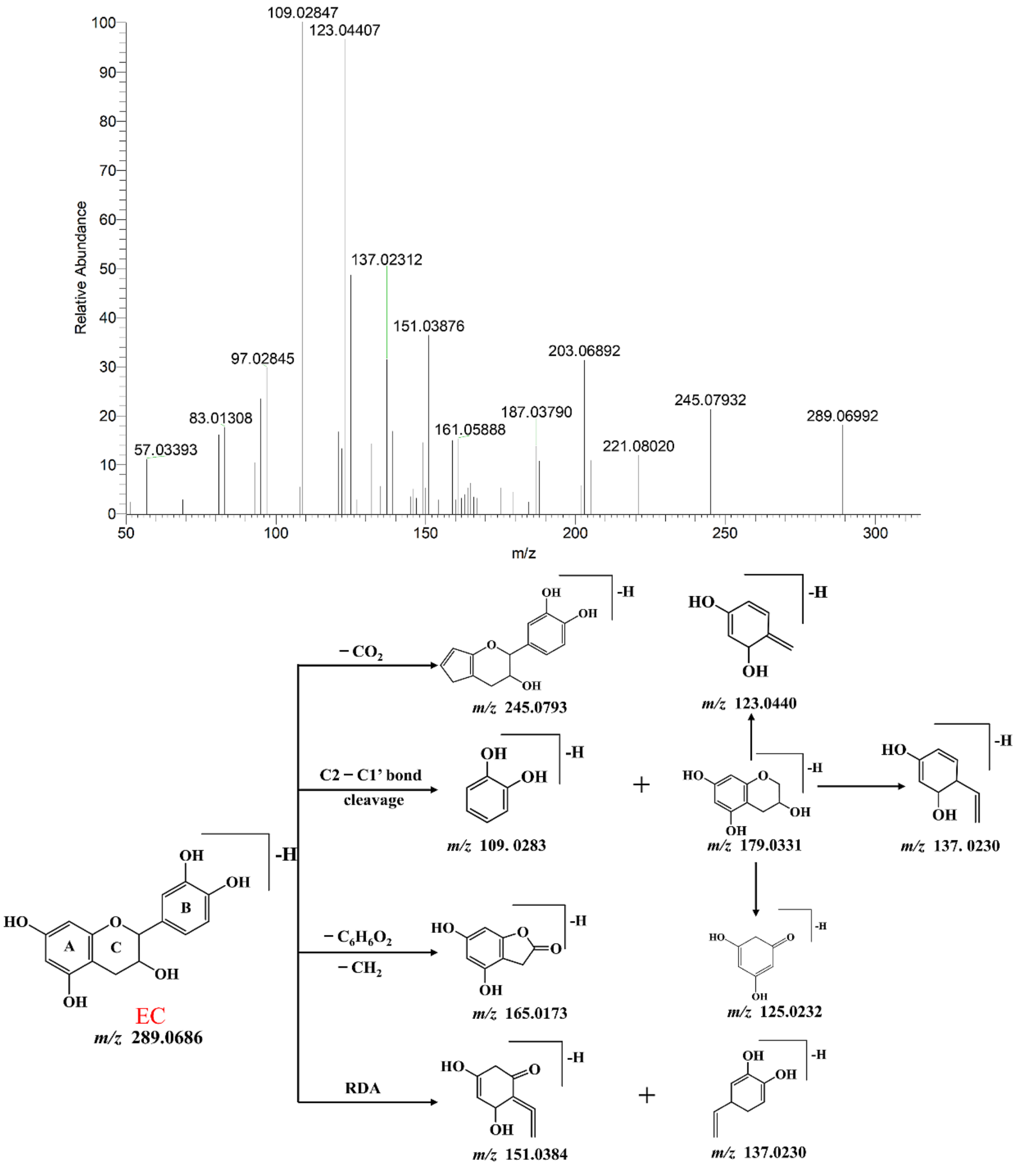

Figure S5. The MS² spectrometry of EC and its cleavage pathway.

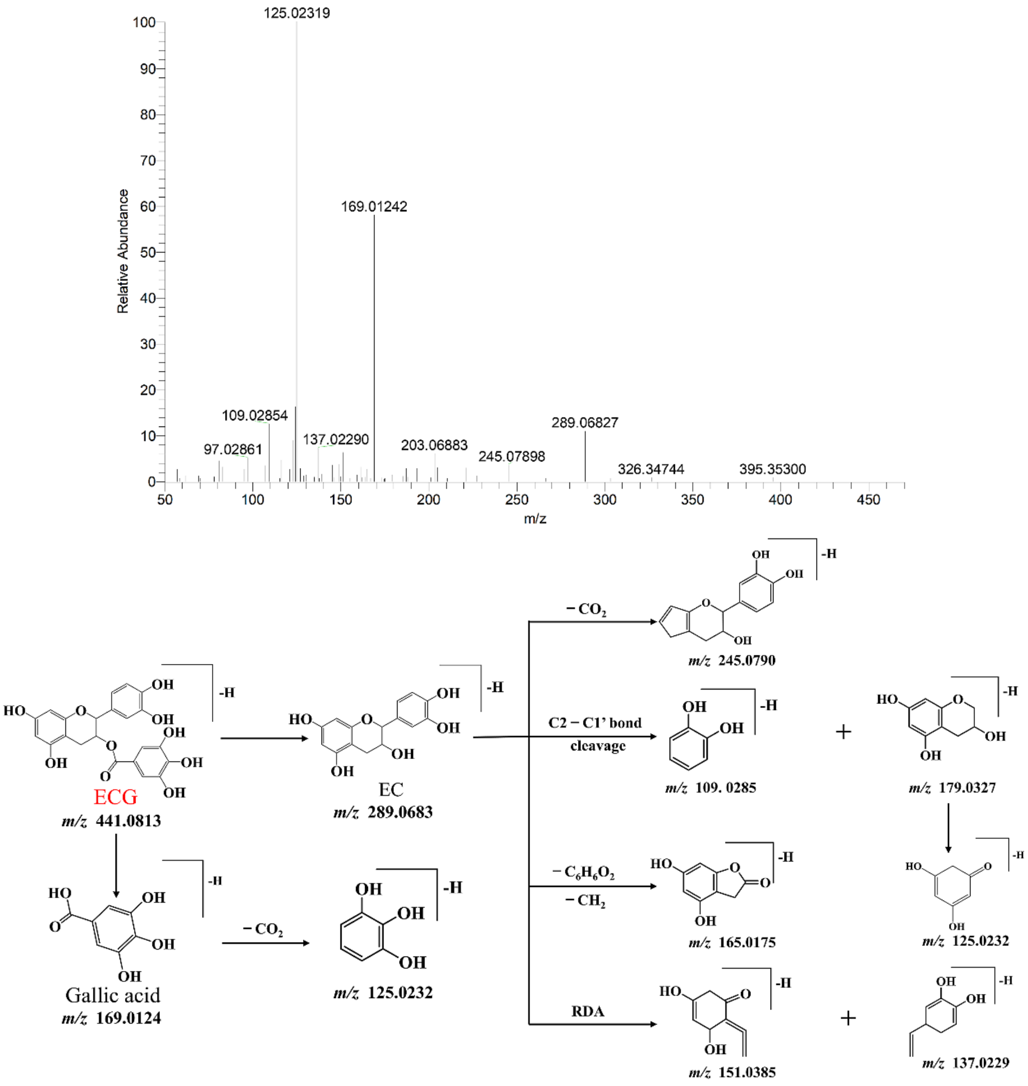

Figure S6. The MS^2 spectrometry of ECG and its cleavage pathway.

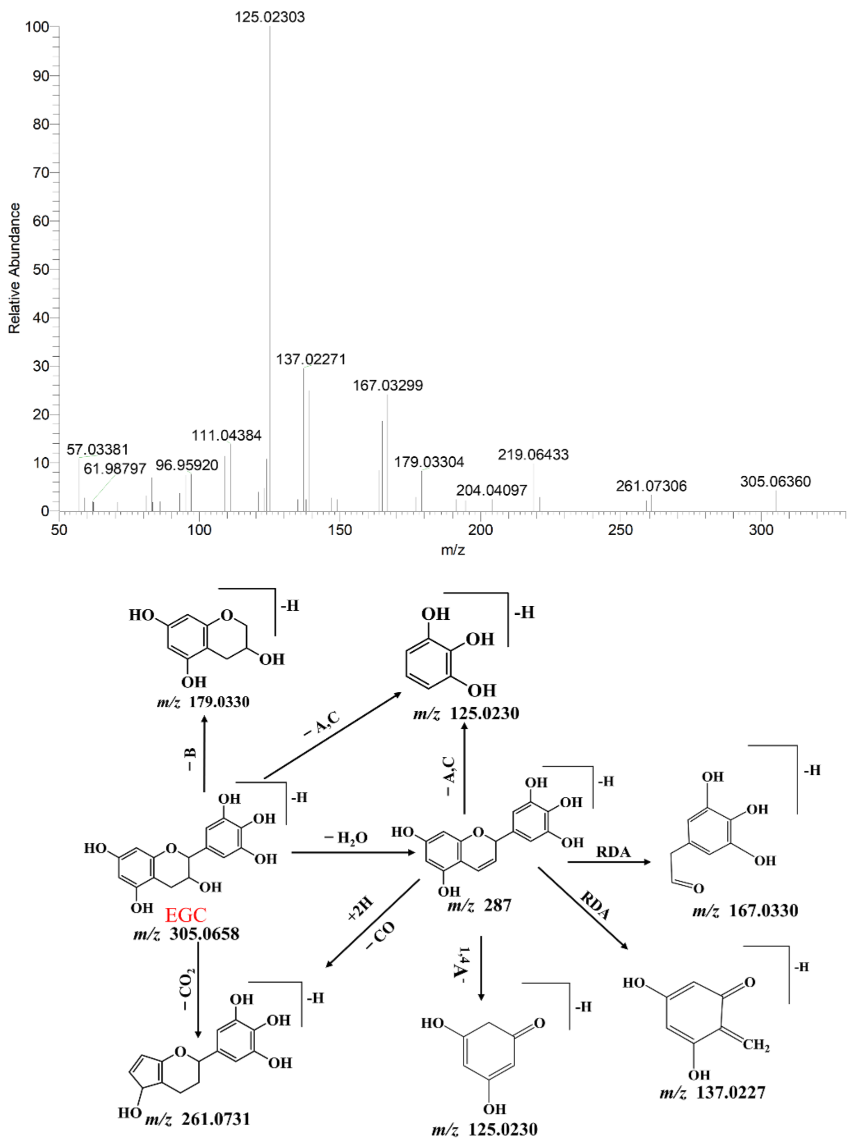

Figure S7. The MS² spectrometry of EGC and its cleavage pathway.

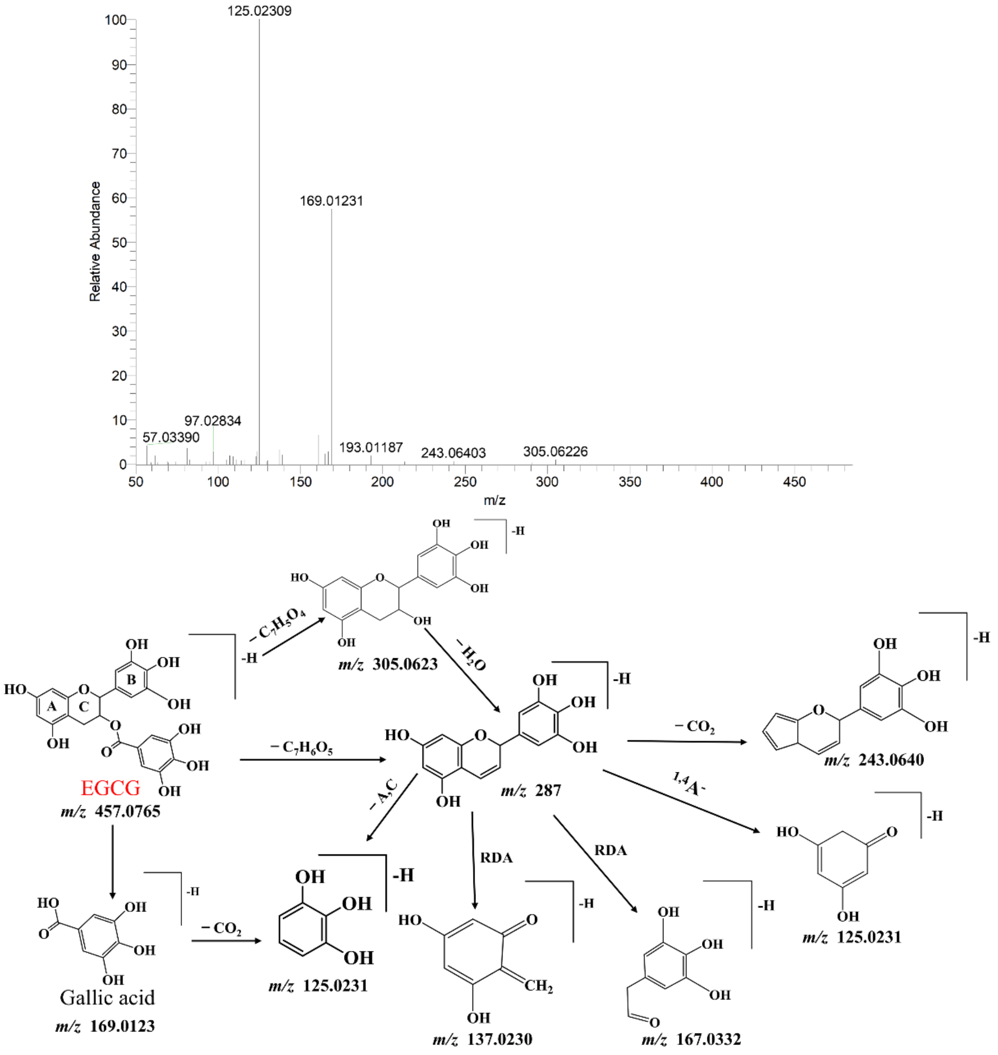

Figure S8. The MS² spectrometry of EGCG and its cleavage pathway.

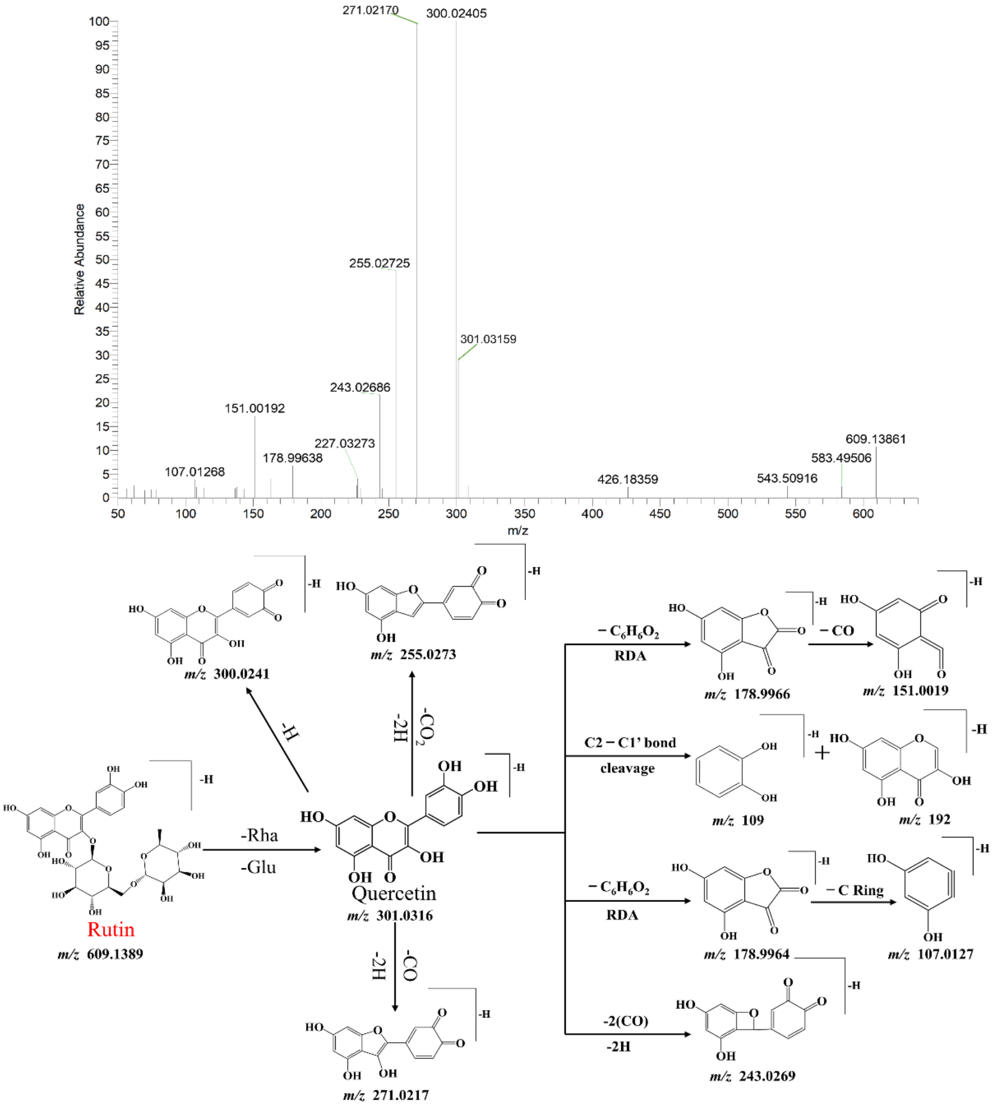

Figure S9. The MS² spectrometry of rutin and its cleavage pathway.

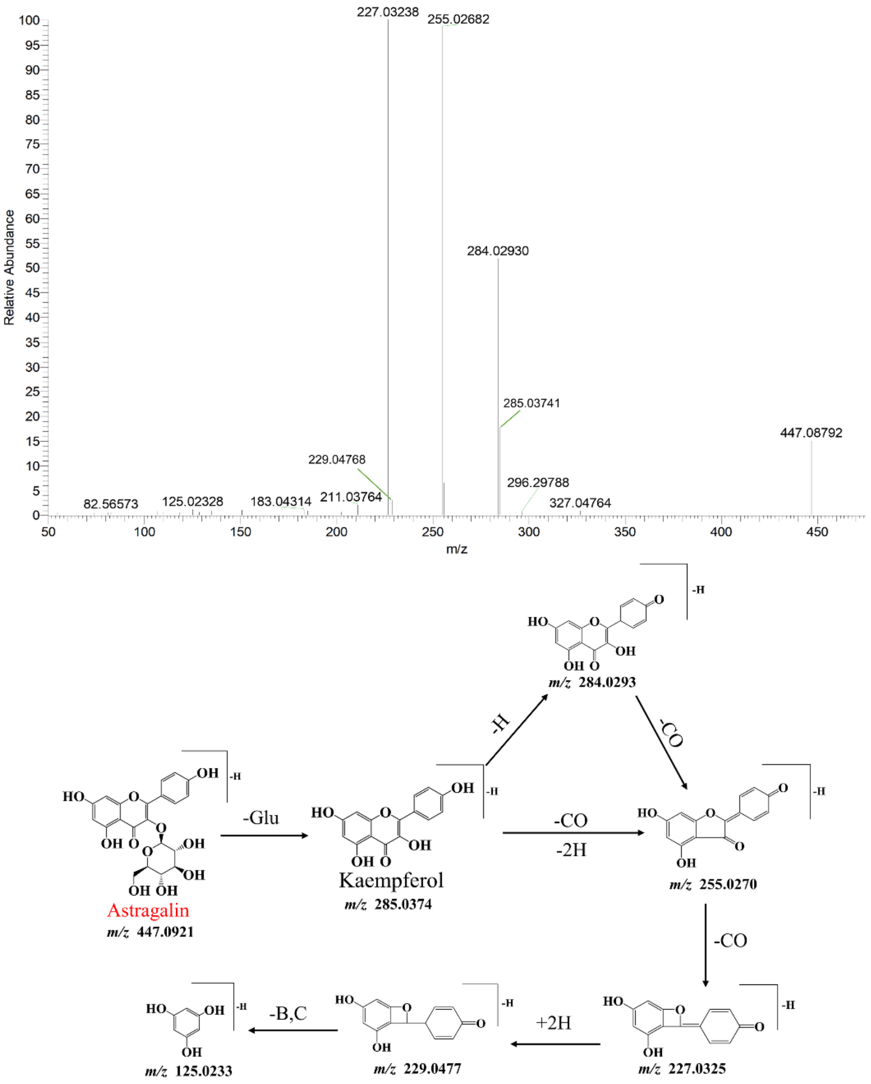

Figure S10. The MS² spectrometry of astragalin and its cleavage pathway.

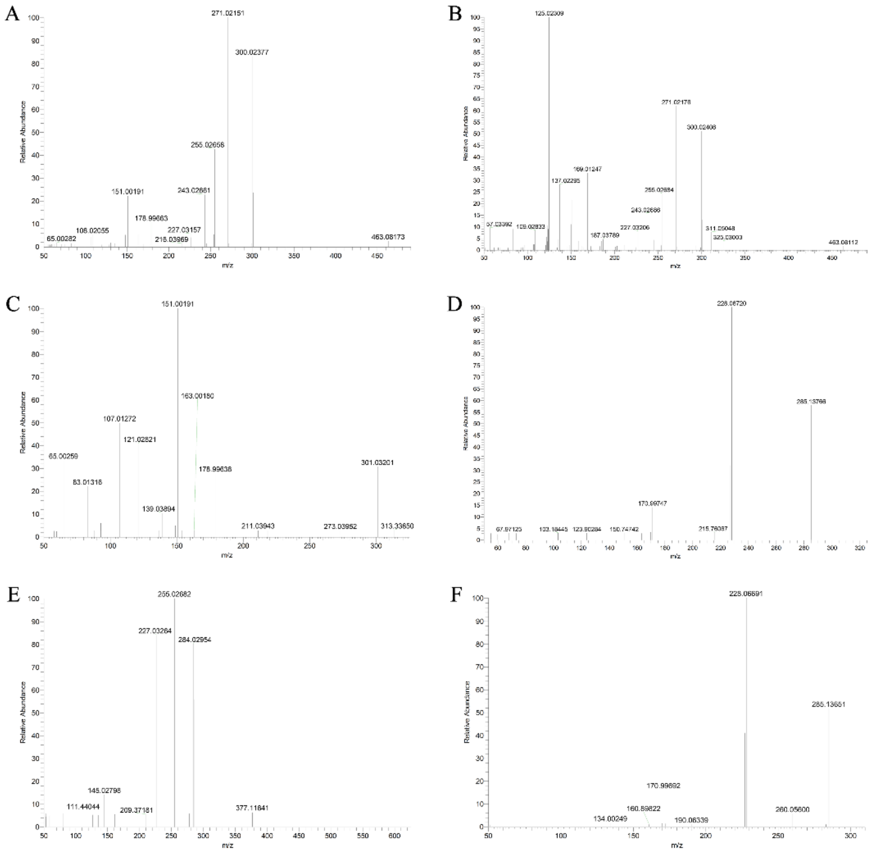

Figure S11. The MS² spectrum of (A) Hyperoside, (B) Isoquercitrin, (C) Quercetin, (D) Taxifolin; (E) Nictoflorin and (F) Kaempferol in GTE.

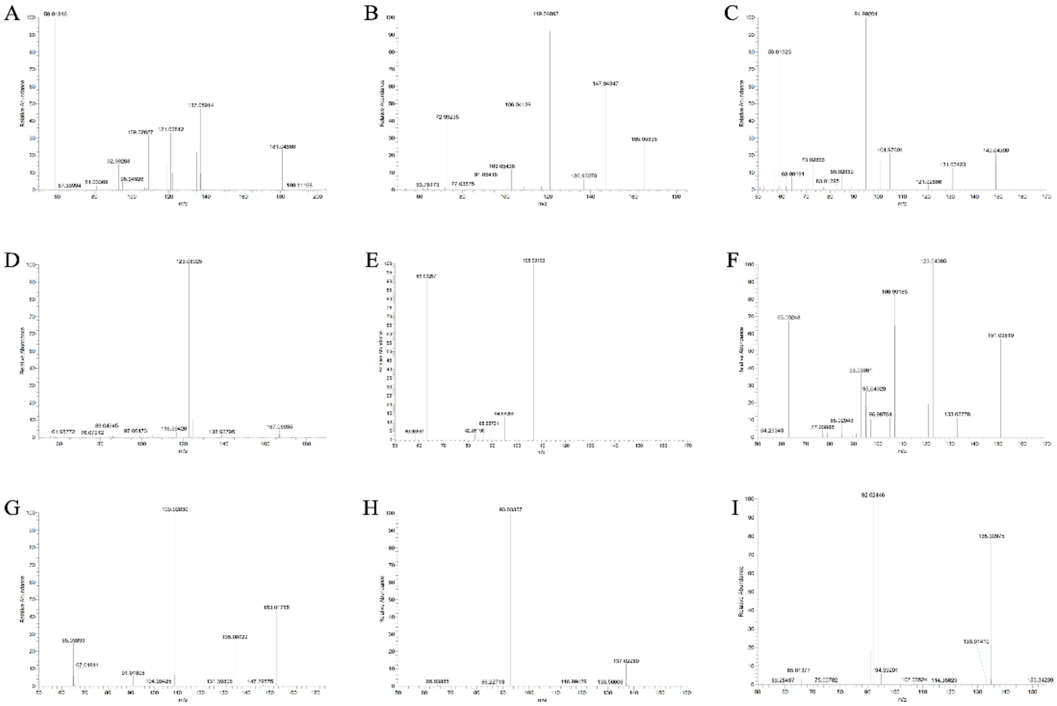

Figure S12. The MS^2 spectrum of (A) M1, (B) M2, (C) M3, (D) M4, (E) M5, (F) M6, (G) M7, (H) M8 and (I) M9.

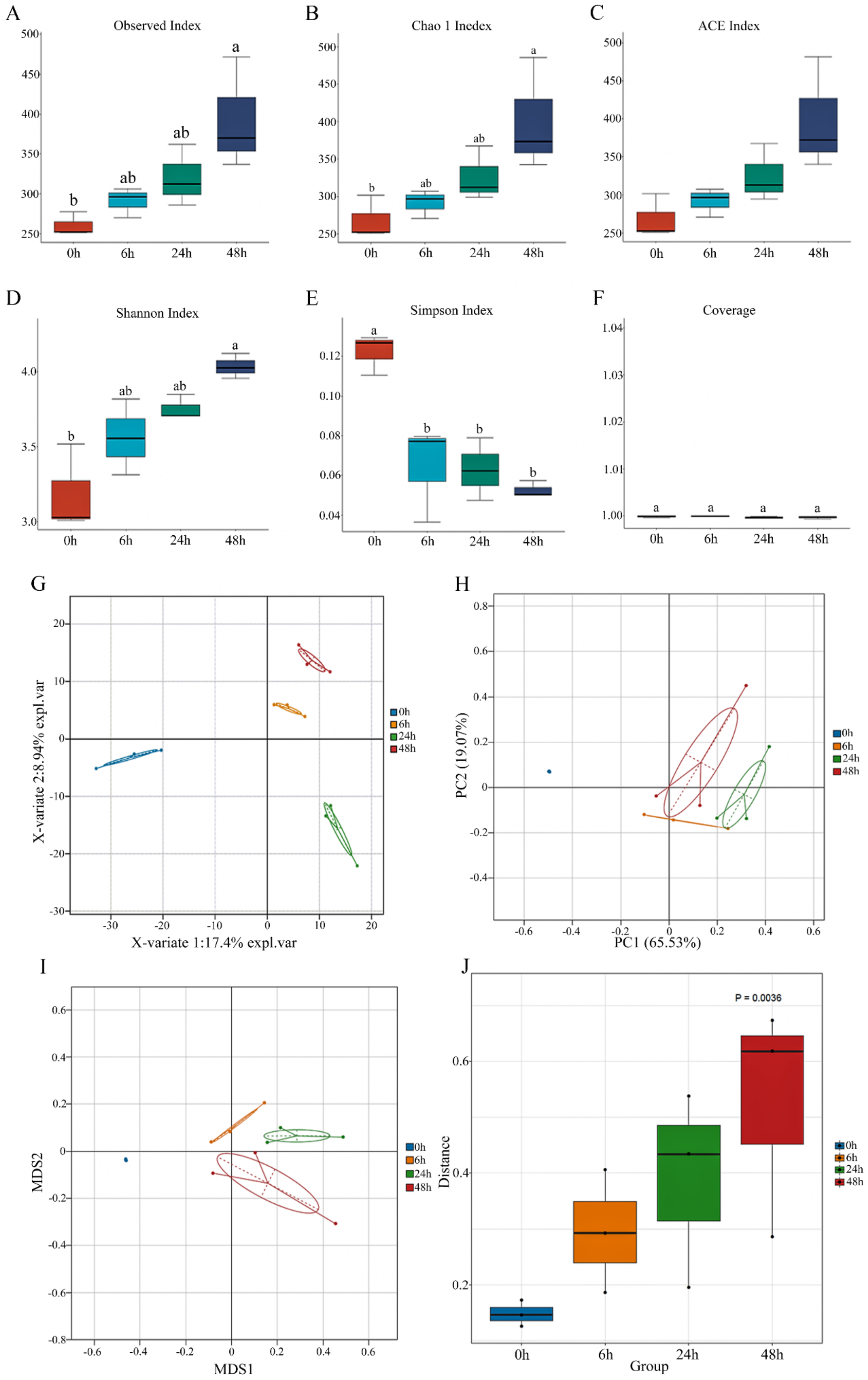

A
Observed Index
B
Chao 1 Inedex
C
ACE Index
D
Shannon Index
E
Simpson Index
F
Coverage
0h
6h
24h
48h
G
X-variate 2:8.94% expl.var
X-variate 1:17.4% expl.var
H
PC2 (19.07%)
PC1 (65.53%)
I
MDS2
MDS1
J
P = 0.0036
Distance
Group

Figure S13. Effects of GTE fermentation time (0h, 6h, 24h and 48h) on the α- and β-diversity of the gut microbiota. (A) Observed species; (B) Chao1 index; (C) ACE index; (D) Shannon index; (E) Simpson index; (F) Goods coverage; (G) PLS-DA score plot; (H) PCoA plot; (I) NMDS plot; (J) PERMANOVA. Different lowercase letters indicate significant differences (n=3, $p < 0.05$).

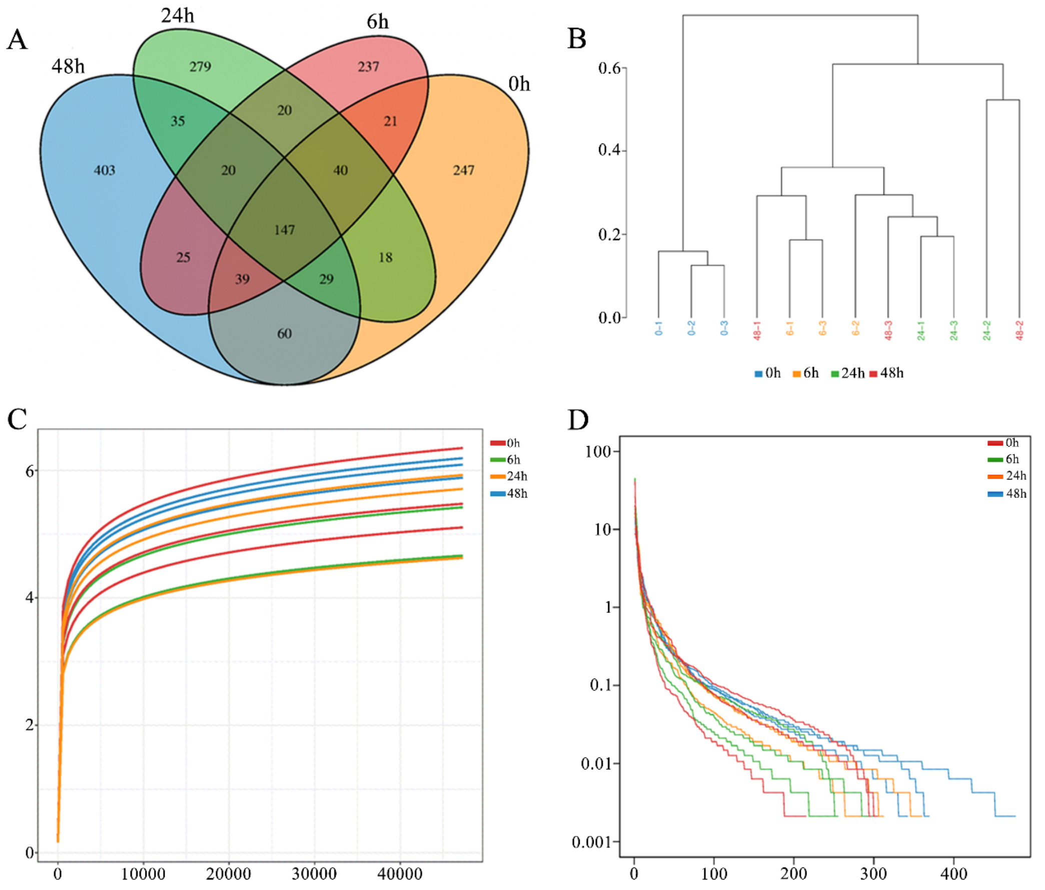

Figure S14. Microbial community analysis of GTE samples during in vitro fermentation via 16S rRNA sequencing. (A) Venn diagram of OTUs/ASVs among samples fermented for 0, 6, 24, and 48 h; (B) Hierarchical clustering of GTE samples across fermentation time points; (C) Shannon–Wiener curves of samples fermented for 0, 6, 24, and 48 h; (D) Rank-abundance curves of samples fermented for 0, 6, 24, and 48 h.

Table S1. Basic information of voluntary donors

Donor Number	Gender	Age	BMI (kg/m²)
1	male	23	21.37
2	male	23	22.64
3	male	24	22.64
4	male	21	20.24
5	female	22	19.23
6	female	22	21.30
7	female	23	19.98
8	female	24	20.20

Table S2. Relative average contents of total antioxidant activity, total polyphenols and total flavonoids in GTE at different fermentation time points (n=3).

Fermentation Time（h）	0	3	6	12	24	48
Relative average content of total antioxidant activity (mg·TE/mL)	0.5877	0.6652	0.6930	0.6356	0.6043	0.5970
Relative average content of total polyphenols (mg·GAE/mL)	0.9588	1.0778	1.1626	1.0815	1.0344	1.0143
Relative average content of total flavonoids (mg·RE/mL)	1.2095	1.1961	1.3222	1.1442	1.1353	1.1338
